# Kinetic principles of ParA2-ATP cycling guide dynamic subcellular localizations in *Vibrio cholerae*

**DOI:** 10.1093/nar/gkad321

**Published:** 2023-05-04

**Authors:** Satpal S Chodha, Adam C Brooks, Peter J Davis, Revathy Ramachandran, Dhruba K Chattoraj, Ling Chin Hwang

**Affiliations:** Department of Molecular Biology and Biotechnology, University of Sheffield, Firth Court, Western Bank, Sheffield S10 2TN, UK; Department of Molecular Biology and Biotechnology, University of Sheffield, Firth Court, Western Bank, Sheffield S10 2TN, UK; Department of Molecular Biology and Biotechnology, University of Sheffield, Firth Court, Western Bank, Sheffield S10 2TN, UK; Basic Research Laboratory, Centre for Cancer Research, National Cancer Institute, National Institutes of Health, Bethesda, MD 20892-4260, USA; Basic Research Laboratory, Centre for Cancer Research, National Cancer Institute, National Institutes of Health, Bethesda, MD 20892-4260, USA; Department of Molecular Biology and Biotechnology, University of Sheffield, Firth Court, Western Bank, Sheffield S10 2TN, UK; Medical Technology Research Centre, School of Medicine, Faculty of Health, Education, Medicine & Social Care, Anglia Ruskin University, Chelmsford, UK

## Abstract

Dynamic protein gradients are exploited for the spatial organization and segregation of replicated chromosomes. However, mechanisms of protein gradient formation and how that spatially organizes chromosomes remain poorly understood. Here, we have determined the kinetic principles of subcellular localizations of ParA2 ATPase, an essential spatial regulator of chromosome 2 segregation in the multichromosome bacterium, *Vibrio cholerae*. We found that ParA2 gradients self-organize in *V. cholerae* cells into dynamic pole-to-pole oscillations. We examined the ParA2 ATPase cycle and ParA2 interactions with ParB2 and DNA. *In vitro*, ParA2-ATP dimers undergo a rate-limiting conformational switch, catalysed by DNA to achieve DNA-binding competence. This active ParA2 state loads onto DNA cooperatively as higher order oligomers. Our results indicate that the midcell localization of ParB2-*parS2* complexes stimulate ATP hydrolysis and ParA2 release from the nucleoid, generating an asymmetric ParA2 gradient with maximal concentration toward the poles. This rapid dissociation coupled with slow nucleotide exchange and conformational switch provides for a temporal lag that allows the redistribution of ParA2 to the opposite pole for nucleoid reattachment. Based on our data, we propose a ‘Tug-of-war’ model that uses dynamic oscillations of ParA2 to spatially regulate symmetric segregation and positioning of bacterial chromosomes.

## Graphical Abstract

**Graphical Abstract:**
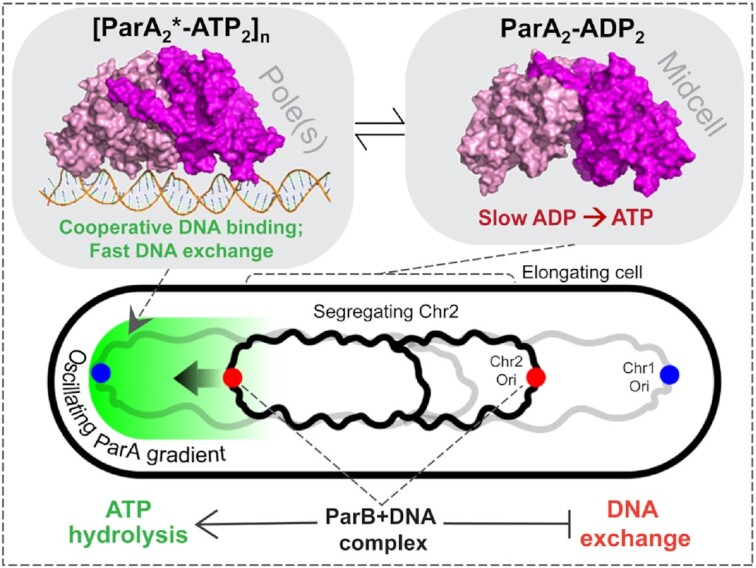
ParA2 gradient oscillates from pole-to-pole to symmetrically segregate the sister loci of Vibrio cholerae Chromosome 2 via Tug-of-war model.

## INTRODUCTION

Chromosome segregation requires precise positioning of replicated chromosomes to opposite cell halves for inheritance of the genetic material. Eukaryotic cells use the spindle apparatus comprising polymerizing microtubules to pull sister chromatids apart during mitosis. Despite the lack of cytoskeletal structure in bacteria, chromosomes are spatially organized in defined patterns with specific segregation dynamics. Most bacteria harness a tripartite ParABS apparatus for chromosome segregation (1–3). First characterized in plasmid partitioning, the ParABS (Par) system consists of centromere-analogous *parS* sequences that reside proximal to the replication origin. ParB proteins bind specifically onto *parS* sites and spread to form the partition complex, which marks the chromosome (cargo) for segregation. ParA ATPase binds to non-specific DNA and via ATP hydrolysis provides for the motive force to position the cargo. Par proteins also contribute to other key cellular processes including DNA condensation, chromosome replication, transcription regulation, cell division and motility (4–8). In bacteria with multipartite genomes such as *Vibrio cholerae* and *Burkholderia cenocepacia*, the Par system is essential for their survival and pathogenesis (9,10).

The ParA/MinD superfamily of P-loop ATPases (deviant Walker ATPases) are involved in gradient formation in many bacteria (11–13). ParA/MinD proteins self-organize to regulate the positioning of macromolecular complexes that are vital for DNA segregation and cell division. These include chromosome and plasmid segregation driven by propagating ParA gradients (14–19), positioning of cell division site by pole-to-pole oscillations of MinCDE in *Escherichia coli* (20,21), bipolar gradient formation of *Caulobacter crescentus* MipZ (22,23), flux-based translocation of *Myxococcus xanthus* PomXYZ (24) and carboxysomes positioning by McdAB in cyanobacteria (25). Despite diverse modes of ParA dynamic gradients, the mechanism of gradient-driven intracellular transport remains enigmatic. Investigating the ParA ATPase cycle and its control by cofactors is the key to understand how ParA proteins have evolved to spatially organize various cellular complexes in different bacteria.

Initial models of plasmid or chromosome segregation were based on *in vivo* studies where ParA clouds appeared as helical filaments pushing or pulling partition complexes apart (14,15,26,27). This was supported by imaging of negatively-stained ParA fibre bundles by electron microscopy (27–30). Biochemical studies of ParA–DNA interactions and cell-free reconstitutions of the P1 and F plasmid partition systems have shifted the paradigm towards a diffusion-ratchet mechanism (31–34). The diffusion-ratchet model is based on ParA gradients providing a chemophoretic force, where collective ParA–ParB tethers drive the processive motion of plasmids on the nucleoid (35,36). Variations of the diffusion-ratchet model have been reported in more recent studies on chromosome and plasmid segregation. *C. crescentus* was proposed to utilise the intrinsic elastic properties of the chromosome to relay the partition complex across the cell (17,37). *B. subtilis* chromosomal loci and F plasmid hitch-hike on ParA bound to high-density regions within the nucleoid (18). Whilst plasmid TP228 has merged both concepts where ParF (ParA) polymers assemble into a 3D meshwork in which the plasmid is trapped and distributed (19,38). However, the underlying principles of ParA gradient formation and how the spatial organization of ParA gradients mediate chromosome segregation, particularly in bacteria with multiple chromosomes, remain poorly understood.

*Vibrio cholerae*, the causative agent for cholera, has its genome split between a larger 3 Mb chromosome 1 (Chr1) and a plasmid-like 1 Mb chromosome 2 (Chr2). Each chromosome encodes its own ParABS system that functions in a chromosome-specific manner. The two Par systems display distinct and independent *in vivo* dynamics (6,13,14). Upon replication, the two Chr1 origins are distributed asymmetrically; one origin travels from the old to the new pole and the other stays at the old pole. When two-thirds of the Chr1 replication cycle is completed, then only Chr2 replication initiates (39,40). The two Chr2 origins then relocate symmetrically from the mid-cell to quarter-cell positions (14,41,42). Migration of the origins of Chr1 and Chr2 are directed by the partition complex, consisting of ParB-bound *parS* sites close to the origin (43). Chr1 ParA1 forms a retracting cloud, pulling one of the ParB1-*parS* loci toward the new cell pole (14,44). This mimics a mitotic-like model where ParA1 filaments polymerize to the sister ParB1-*parS* complexes and retract to pull them apart (14). The ParABS1 system is not essential for Chr1 segregation, which employs an unknown mechanism for segregation in absence of Par system (43,45). On the contrary, the ParABS2 system is essential for Chr2 segregation and *V. cholerae* viability. Deletion of *parAB2* loci results in loss of Chr2 over time and activation of toxin-antitoxin systems that kill cells, thereby causing loss of *V. cholerae* pathogenesis (9). ParA2 was initially shown by negative staining to form a left-handed helical filament on DNA (46). More recently the crystal structures of ParA2 apo and ADP-bound states, and the cryo-EM structure of ParA2 filament bound to DNA have been solved (47). ParA2 dimers undergo a dramatic structural rearrangement upon DNA binding that exposes an oligomerization interface, allowing it to form filaments. These studies provide a structural insight into how high-density ParA bound regions form on the nucleoid. There has been no reported ParB2 structures to date, although recent ParB structures from *Bacillus subtilis* and *Myxococcus xanthus* offer clues to the architecture of partition complexes (48–50). Despite the vital role of ParA2 in *V. cholerae* proliferation, very little is known about its subcellular dynamics and biochemical properties.

Here, we found that ParA2 forms asymmetric gradient that dynamically oscillates in *V. cholerae* cells. *In vitro*, ParA2 dimers upon binding ATP, undergo a rate-limiting conformational switch that is catalysed by DNA to achieve DNA-binding competence. This activated ParA2 binds DNA cooperatively as higher-order oligomers to dynamically pattern the nucleoid. Nucleotide exchange is also slow, which might be controlling the rate of DNA rebinding and gradient formation. Nonetheless, the ATP cycling and DNA rebinding rates of ParA2 were significantly faster compared to those of plasmid ParAs. Based on these findings, we propose a ‘Tug-of-war’ model that uses ParA2 dynamic oscillations to regulate the symmetrical positioning and segregation of a large chromosomal cargo that is more responsive in coordinating segregation dynamics with cell division.

## MATERIALS AND METHODS

### Buffers

**Buffer A**: 50 mM Tris–HCl pH 7.5, 100 mM NaCl, 10 mM MgCl_2_, 10% glycerol, 100 mg/ml BSA, and 1 mM DTT. **Buffer B**: 50 mM Tris–HCl pH 7.5, 150 mM NaCl, 5 mM MgCl_2_.

### Microscopy of ParA2 in *V. cholerae*

Cells were grown in 1× M63 medium supplemented with 1 mM CaCl_2_, 1 mM MgSO_4_, 0.001% vitamin B1, 0.2% fructose, 0.1% casamino acids at 30°C until an OD_600 nm_ of 0.3. When required, kanamycin was added to a final concentration of 12.5 μg/ml. Expression of ParA2:GFP or ParA2 K124X:GFP (where X = Q, E or R) was induced by adding 0.0008% arabinose for 1 h at 30°C with shaking. 10 μl of the culture was plated on the centre of a glass P35 dish (MatTek corporation, Ashland, MA), and overlaid with a 1% agarose disc prepared with the same M63 medium described above but supplemented with 0.02% arabinose. Images were taken every 30 secs on a Nikon Ti-Eclipse inverted microscope with Nikon 100×/1.4 Oil Plan Apo Ph3 DM objectives, imageEM EMCCD camera (Hamamatsu, Japan) and Lumencor sola light engine (Beaverton, OR) set to 5% 475 nm laser output and 300 ms exposure.

### SEC-MALS

Samples of 40 μM ParA2 were incubated alone, or in the presence of 1 mM ATP or ADP, in 50 mM Tris–HCl pH 7.5+(210 mM NaCl, 5.0 mM MgCl_2_, 0.1 mM EDTA, 1.0 mM DTT, 1.0 mM NaN_3_), for 20 min at 37°C. SEC-MALS of ParA2 was performed with 15 μl injections into a GE Superdex 200 10/300 GL SEC column at 0.75 ml/min equilibrated and run in 50 mM Tris–HCl pH 7.5 buffer+(100 mM NaCl, 5.0 mM MgCl_2_, 0.1 mM EDTA, 1.0 mM DTT, 1.0 mM NaN_3_) using a Postnova AF2000 system with PN5300 autosampler. Protein elution was monitored with a Shimadzu Prominence SPD-20AV (PN3212) UV absorbance detector, PN3621b MALS detector and PN3150 Refractive Index Detector. Data analysis was conducted with NovaFFF AF2000 2.1.0.1 (Postnova Analytics, UK Ltd) software and values plotted in Graphpad Prism 8.0.2. For protein concentration determination, a UV_280 nm_ molar extinction coefficient of 1.03 M^−1^cm^−1^ was used and absolute molecular weights were calculated using Zimm fits. Data was averaged from three repeat measurements.

### ATPase activity

For pre-steady state ATPase activity measurements, 1.5 μM ParA2 or its mutant derivatives, 100 μM ATP and 64 nM [α-^32^P]ATP were incubated in Buffer A. Where indicated, 1.5 μM ParB2 and/or 0.1 mg/ml sonicated salmon sperm DNA were added. 10 μl reactions were assembled on ice, incubated for the indicated time periods at 37°C and quenched by the addition of 10 μl 1% SDS and 20 mM EDTA. For steady state activity assays, indicated concentrations of ParA2 were incubated in reactions set up as described above, at 37°C for 30 min. 1 μl from each sample was spotted onto a POLYGRAM CEL 300 PEI-TLC plate (Macherey-Nagel), and developed with 0.5 M LiCl (Sigma) and 1 M formic acid (Alfa Aeser). Dried plates were exposed to a storage Phosphor screen and scanned with a phosphoimager (Typhoon FLA7000 IP) for quantification using ImageJ (NIH).

### Circular dichroism spectroscopy (CD)

CD experiments were performed in filtered and degassed buffer containing 10 mM Tris pH 8.0+5 mM MgCl_2_. Reaction mixtures were prepared with 5 μM ParA2 and 2 mM of either ATP, ADP, AMPPNP, ATPγS, or no nucleotide. ParA2 with ATP in the absence of MgCl_2_ was prepared in 10 mM Tris pH 8.0+2 mM EDTA. The samples were filtered by centrifugation using a 0.2 μm Generon Proteus Clarification Mini Spin Column (GENMSF-500). The reactions were incubated at 23°C for 15 min. Spectra were measured using a Jasco J-810 Spectropolarimeter in a 1 mm Hellma Analytics QS High Precision Cell. Measurements were collected from 300 to 200±2.5 nm, in 1 nm intervals with an 8 s integration time. Blank buffer solutions containing corresponding nucleotides were subtracted from the ParA2 spectra. Each experiment was repeated at least twice and each spectra is an average of 3 scans. ParA2 secondary conformation was monitored by CD at 220±2.5 nm with 8 s integration time, from 23°C to 63°C. The temperature was increased in 2°C increments, and the sample was equilibrated to each temperature for 1 min before measurement of the signal.

### Nucleotide binding, dissociation and exchange assays

Stopped flow measurements with MANT (*N*-methylanthraniloyl)-labeled nucleotides (Jena) were performed at 23°C using Applied Photophysics SX20. The excitation monochromator wavelength was 356±1.2 nm and emission filter was BLP01-405R-25 (Semrock). Nucleotide binding, dissociation, and exchange experiments were performed in Buffer B with samples prepared on ice. For nucleotide binding assays, 0.6, 1.25 or 2.5 μM ParA2 was rapidly mixed with 25 μM MANT-AXP (AXP = ATP or ADP) and fluorescence increase was monitored over time. For pseudo-first order reaction, 0.3125, 6.25, 1.25 or 2.5 μM ParA2 was rapidly mixed with 3.125, 6.25, 12.5 or 25 μM MANT-AXP in buffer B and their fluorescence increase monitored. The observed binding curves were fitted with single exponential increase to determine observed rate of binding, *k*_obs_. Plots of *k*_obs_ versus substrate concentration yielded *k*_on_ and *k*_off_ from the slopes and y-intercepts, respectively (51). For nucleotide dissociation assay, 2.5 μM ParA2 and 5 μM MANT-AXP were pre-incubated at 23°C for 3 min, then rapidly mixed with 1 mM unlabelled AXP and their fluorescence decrease monitored. For nucleotide exchange assay, 0.625, 1.25 or 2.5 μM ParA2 was pre-incubated at a 1:5 ratio with 3.125, 6.25 or 12.5 μM unlabelled AXP, respectively, then rapidly mixed with 15.625, 31.25 or 62.5 μM MANT-AXP at 5× higher concentrations than AXP. All data were averages of at least two experiments. Values were reported as relative fluorescence increase or decrease.

### Tryptophan fluorescence experiment

For steady state reactions, 0.6 μM ParA2 with 1 mM ATP, ADP or ATPγS were incubated at 23°C for 15 min in Buffer B. In the absence of MgCl_2_, a separate buffer was prepared with 0.1 mM EDTA and without MgCl_2_. Tryptophan fluorescence signal was acquired using a SpectraACQ spectrafluorimeter set at 356±1.2 nm. FluorEssence V3.5 software was used for plotting data and GraphPad Prism for data analysis. Stopped-flow measurements were performed at 23°C using Applied Photophysics SX20 system. The excitation monochromator wavelength was 295 nm and emission filter was BLP01-325R-25 (Semrock). For kinetics experiment, 1.2 μM ParA2 was rapidly mixed with 2 mM MANT-AXP in Buffer B and when present, 0.2 mg/ml DNA and 1.2 μM ParB2. Final concentrations after mixing are half of initial concentrations. All results are averages of at least two experiments. Values were reported as relative fluorescence increase or decrease.

### EMSA

A standard reaction mixture (20 μl) was prepared in Buffer A with 5 nM Cy3-labeled 69 bp DNA and 2 mM of ATP, ADP, ATPγS or no nucleotide, with increasing concentrations of ParA2 as indicated. The reactions were assembled on ice, incubated for 30 min at 30°C, and analysed by electrophoresis in 5% polyacrylamide gels in TBM (90 mM Tris, 150 mM Borate, 10 mM MgCl_2_). Gel was pre-run at 120 V for 30 min at 4°C, in a Mini-PROTEAN Tetra Cell, and then run at 120 V for 1 h at 4°C. Gels were imaged using a Bio-Rad ChemiDoc™ MP Imaging System using the Cy3 channel with 2 min exposure. Images were analysed with ImageJ (NIH).

### TIRF microscopy

A home-built prism-based TIRF microscope was set up using a Ti-Eclipse (Nikon) microscope with a PlanApo 100x NA 1.45 oil-immersion objective. Laser excitation light of 488 nm (Cobolt 06-MLD) at laser power 100 μW was focused and aligned onto a prism placed on a quartz slide for TIRF illumination at the centre of the objective. The fluorescence emission light was filtered by a notch filter (Thorlabs NF488-15) and bandpass filter (Chroma ET535/70m). Fluorescence images were captured using a sCMOS camera (Prime 95B, Photometrics) with exposure time 100 ms, frame rate 1 s at 16-bit depth. The camera bias of 100 arbitrary units was subtracted from measured intensity. Micro-manager software was used for camera control and image acquisition. ImageJ (NIH) was used for image analysis. Syringe pumps (WPI) were controlled using RealTerm open software.

### Flowcell assembly

Single-inlet and dual-inlet flowcells with Y-shaped channel were assembled and coated with biotinylated lipid bilayer, neutravidin and DNA carpet as previously described (32,33).

### ParA2-GFP binding and dissociation on DNA carpet

ParA2-GFP (10 μM) was preincubated in Par Buffer (50 mM Tris pH 7.5, 100 mM NaCl, 5 mM MgCl_2_, 10% (v/v) glycerol, 1 mM DTT and 0.1 mg/ml α-casein) with 1 mM ATP, ADP or ATPγS for 30 min at 25°C. The sample was diluted to final protein concentrations as indicated with Par buffer. The sample was loaded into a 1 ml syringe (BD) and attached to inlet 1 of a dual-inlet flowcell. A separate syringe containing wash buffer (Par Buffer) was attached to inlet 2. The TIRF illumination field and microscope objectives were aligned to the Y-channel junction at the point of flow convergence. For ParA-GFP binding, sample and wash buffers were infused simultaneously into the flowcell at 20 μl/min and 1 μl/min respectively. After 380 s, sample and wash buffer flow rates were switched to 1 μl/min and 20 μl/min respectively, for protein dissociation. Data was analysed using GraphPad Prism 7. Binding curves were fitted with ‘one phase association’ model whilst dissociation curves were fitted with a ‘two phase decay’ model.

### Additional procedures

For plasmid construction and protein purification, see Supplementary Data.

## RESULTS

### ParA2 displays dynamic localization in *V. cholerae*

**Figure 1.**
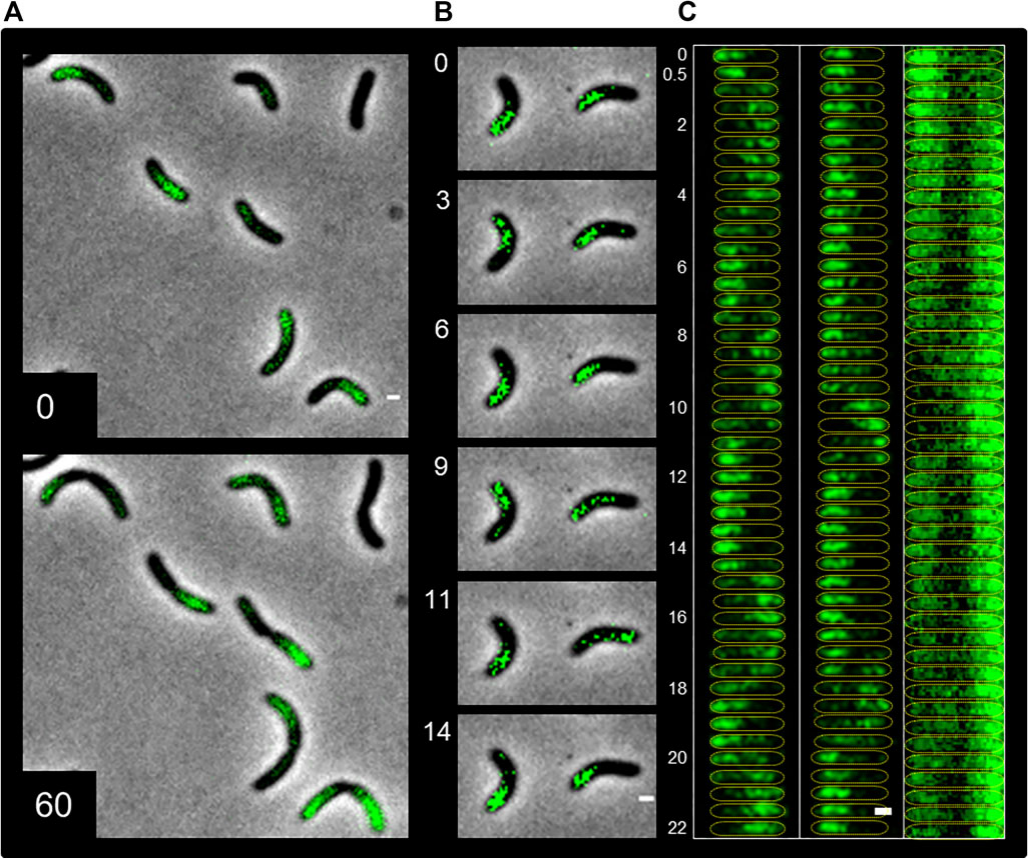
Dynamic localization of ParA2-GFP in *V. cholerae* cells. Representative cells carrying plasmids expressing ParA2-GFP. (**A**) A population of cells displaying asymmetric gradients of ParA2-GFP (green) from cell poles at 0 min (top) and after 60 min (bottom). (**B**) Time-lapse imaging of two cells showing pole-to-pole oscillations of ParA2-GFP gradients. (**C**) Kymographs of the left and middle cells from (B, yellow dotted outlines) show periodic ParA2-GFP oscillations with time. Rightmost kymograph shows bipolar ParA2-GFP distribution in a dividing cell and subsequent oscillations between the left cell pole and the forming septa. Numbers are in min and all scale bars represent 1 μm.

To monitor the spatiotemporal distribution of ParA2 in wild type V. cholerae cells, we expressed ParA2 with a C-terminally fused GFP (ParA2-GFP) from an inducible promoter, leaving the native *parA2* locus intact. The expression of the fusion protein affected the cell growth rate (Figure S5A), necessitating controlling the induction level and duration. At the level of expression used, cell-length distribution was not significantly altered, nor did chubby cells appear, indicating stability of Chr2 maintenance (9). We believe at the level of expression used, ParA2-GFP serves as a tracer for WT ParA2, since subcellular dynamics was seen in at least 50% of the cells by time-lapse microcopy, as we discuss below. We observed that almost all the cells that expressed ParA2-GFP had an asymmetric distribution of the protein along the long-axis of the cell (98%, *n* = 61) (Figure 1A). The distribution showed an asymmetric gradient with the highest intensity toward one of the cell poles that gradually decreased as it approached the cell center in predivisional cells, or to the septum in dividing cells. In time-lapse imaging, the ParA2-GFP gradients showed variability in the spatiotemporal dynamics. About 50% of the cell population showed ParA2-GFP gradients either persisting at the same location or migrating to the opposite pole once in the same cell cycle (Figure 1A). On the other hand, the remaining 50% of the cells showed more dynamic localizations of ParA2-GFP, a typical example of which is shown in Figure 1B (Movie S1). In these cells, ParA2-GFP gradients started from one pole and transitioned to form the gradient at the opposite pole before switching back again. Several periods of pole-to-pole oscillation occurred during each cell cycle. Kymographs of the cells show their oscillation periods varied from 4 to 6 min and pole-to-pole transition times from 30–60 s with longer residence times at the cell poles (Figure 1C, left and middle). In some dividing cells, ParA2-GFP gradients were observed at both the cell poles (Figure 1C, right). In some of these cells, ParA2-GFP began to oscillate between the pole and the closing septum. A representative cell in Figure 1C (right) shows an initial polar ParA2-GFP gradient redistributing to bipolar locations at 2 min, followed by oscillations of ParA2-GFP between the left cell pole and the dividing septa at 10 min and 18 min. While oscillatory dynamics are also exhibited by MinD and plasmid ParAs (15,19,20,26,52), periodic oscillations have not been consistently observed for chromosomal ParAs. *In vivo* dynamics of chromosomal ParAs are more varied; *V. cholerae* ParA1 of Chr1 forms a cloud that leads the ParB1-*parS1* foci to the new pole at once per cell cycle, resembling that of *C. crescentus* (27). *B. subtilis* Soj localize at the septa and origin region in its replication-inhibitory state when stimulated by Spo0J, and rebinds the nucleoid during replication initiation (53,54). To understand how ParA2 dynamically localizes in *V. cholerae* and mediates Chr2 segregation, we characterized the biochemical and kinetic properties of interactions involving ParA2.

### ParA2 dimerize without nucleotides

**Figure 2.**
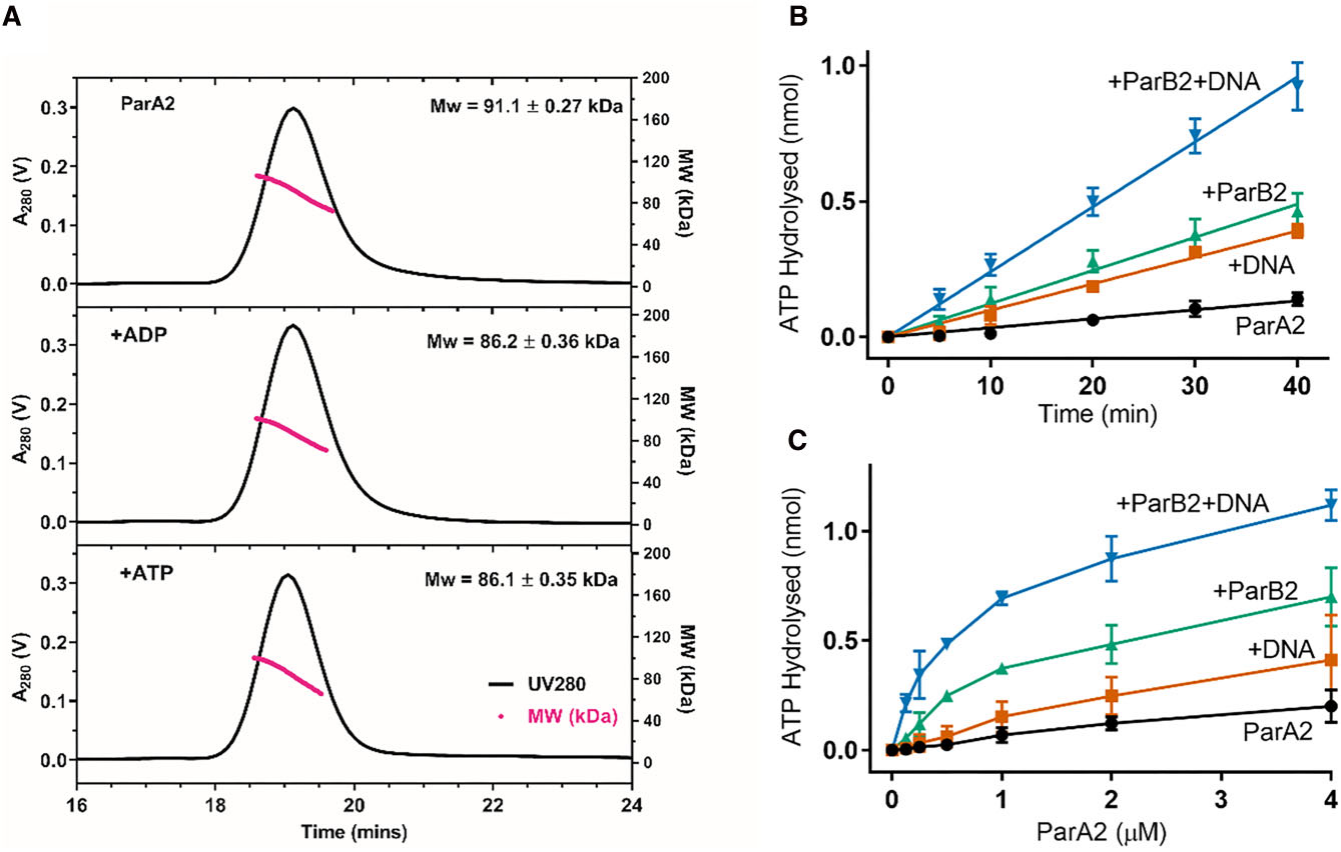
ParA2 forms dimers without ATP and its ATPase activity is stimulated by ParB2 and DNA. (**A**) ParA2 dimerizes with and without adenine nucleotides. SEC-MALS profiles of ParA2 in absence of nucleotide (top), and in the presence of ADP (middle) and ATP (bottom). 40 μM ParA2 was pre-incubated with 2 mM nucleotide for 15 min, when present. Running buffer contained 0.5 mM nucleotide. (**B**) ParA2 pre-steady state ATP hydrolysis kinetics. 1.5 μM ParA2 was mixed in buffer A with 200 μM ATP spiked with 64 nM [α-^32^P]-ATP. 1.5 μM ParB2 and 100 μg ml^−1^ sonicated salmon sperm DNA were added where indicated. The hydrolysis products were measured at 23°C after the indicated reaction times. (**C**) ParA2 steady state ATP hydrolysis. Same as in (B), except that ParA2 concentration was varied as indicated and hydrolysis products were measured after a fixed interval of 30 min.

We purified wild type ParA2 and found it to be stable and soluble for *in vitro* assays (see Supplementary procedures and Figure S1A). To determine the oligomerization state of ParA2 with ATP-binding, we performed size exclusion chromatography multi-angle light scattering (SEC-MALS) analysis. At 40 μM, the majority of the population already formed ParA2 dimers with an average MW of 91.1 kDa (theoretical MW 92.8 kDa) without nucleotides (Figure 2A). The peak positions and widths of elution profiles remained relatively unchanged with ADP and ATP. The very low mean sample polydispersity index of 1.014 across all samples indicates that a single species predominates. The peak MW remained slightly below that expected for a dimer, with a mean equivalent mass of 1.89 monomers. This lower-than-expected mass is most likely the result of a small population (<5%) of monomeric species present in equilibrium with dimers, regardless of the presence of ATP nucleotides. This is in contrast with other plasmid and chromosome ParAs, which exists as monomers with ADP (TP228 ParF) or without ATP (P1 ParA, *C. crescentus* ParA and Soj) (17,31,54–57). P1 ParA when analysed at similar concentrations, is in a monomer-dimer equilibrium without nucleotide and stabilizes as a dimer with ATP or ADP (31). This shows that ParA2 has a higher affinity than P1 ParA to dimerize without nucleotides. The X-ray structures of ParA2 dimer show that each ParA monomer binds 1 nucleotide to form a sandwich dimer (47). From this data, and published data on ParA homologs such as P1/P7 ParA, F SopA and *Hp* Soj we infer that the binding stoichiometry of ParA2 monomer to nucleotide is 1:1.

### ParA2 ATPase activity is stimulated by ParB2 and DNA

ParA2 belongs to the family of P-loop ATPases that contains a deviant Walker A motif (**K**GGTG**K**S) that is involved in ATP-binding and hydrolysis. To determine the ATPase activity of ParA2, we incubated ParA2 with [α^32^P]-ATP and used thin layer chromatography to separate the hydrolyzed products. We found that ParA2 is a weak ATPase with a catalytic constant, *k*_cat_ that is higher than those of plasmid and chromosomal ParA homologs (Figure S1B, C). Similar to plasmid ParAs, its pre-steady state ATP hydrolysis rate was stimulated over 2-fold by nonspecific DNA and 3-fold by its cognate partner, ParB2 at equimolar concentrations (Figures 2B, S1B). The stimulation increased to 8-fold in the presence of both DNA and ParB2. Steady state ATPase activity at increasing ParA2 concentrations showed these pre-steady state rates were close to maximum under these conditions (Figure 2C). The amount of ATP hydrolyzed saturated towards higher ParA2 concentrations due to depletion of [α^32^P]-ATP substrate (Figure 2C). In summary, ParA2 ATPase activity is stimulated by both DNA and ParB2 to a *k*_cat_ level higher than that of other plasmid and chromosomal ParAs (Figure S1B, C, see Discussion).

### ParA2 dimers exhibit slow nucleotide exchange rates

P1 ParA undergoes a slow multi-step conformational change upon ATP binding to switch to a DNA-binding state. This time delay in rebinding once released from DNA, was proposed to generate a ParA gradient surrounding the plasmid, driving plasmid mobility toward higher ParA concentrations (31). To test if *V. cholerae* uses a similar mechanism for ParA2 gradient formation, we investigated the rate-limiting-step in the ATPase cycle of ParA2. Using fluorescently-labelled adenine nucleotides, MANT-ATP and MANT-ADP, we determined their binding affinities to ParA2. At steady state, ParA2 bound MANT-ATP and MANT-ADP similarly with *K*_D_ ∼11 μM (Figure S2). This affinity is 2- and 3-fold higher than that was determined in a previous study on ParA2: 22 μM (ATP) and 34 μM (ADP) (46).

**Figure 3.**
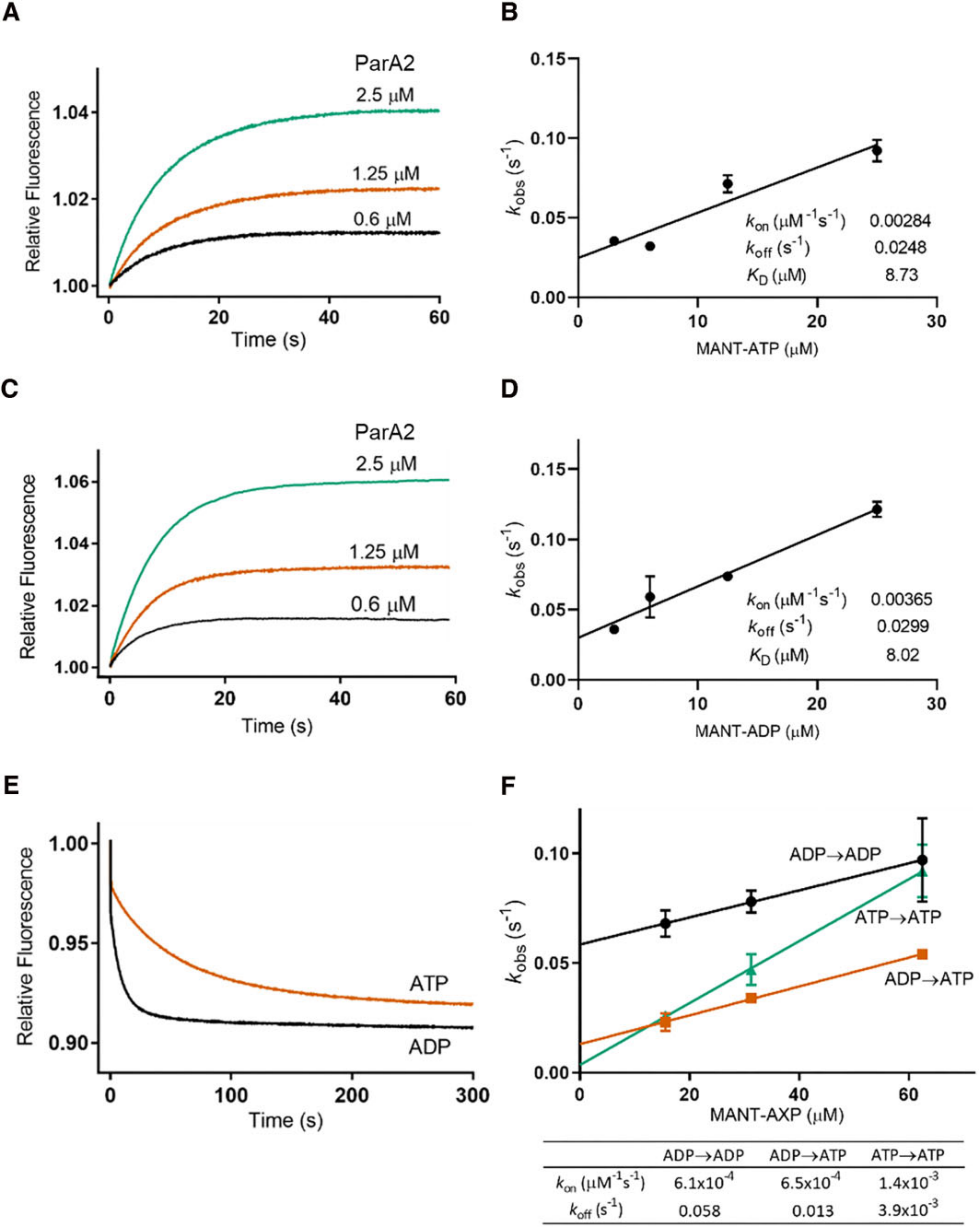
ParA2 dimers exhibit slow nucleotide exchange rates. (**A**) ParA2-MANT-ATP binding kinetics. ParA2 at indicated concentrations and 25 μM MANT-ATP were prepared separately in Buffer B. Stopped-flow fluorescence spectroscopy was used to mix rapidly and monitor the change in relative MANT fluorescence upon ATP binding. (**B**) Plot of pseudo-first order rate constant *k*_obs_ versus MANT-ATP concentration. Samples were prepared as in (A), except MANT-ATP was 10x higher concentration than ParA2. (**C**) ParA2-MANT-ADP binding kinetics. As in (A), except with MANT-ADP. (**D**) Plot of pseudo-first order rate constant *k*_obs_ against MANT-ADP concentration. Samples were prepared as in (C), except MANT-ADP was 10x higher concentration than ParA2. (**E**) ParA2-MANT-AXP (AXP is ATP or ADP) dissociation kinetics. ParA2, at indicated concentrations, was prepared with MANT-AXP in a 1:2 ratio and pre-incubated at 23°C for 3 min, then rapidly mixed with 1 mM AXP. Dissociation kinetics were measured as a decrease in relative MANT fluorescence. (**F**) Nucleotide exchange kinetics of ParA2. ParA2 and unlabelled AXP were pre-incubated to indicated concentrations at a 1:5 ratio, then rapidly mixed with MANT-AXP at a 5× higher concentration than AXP. *k*_obs_ plots versus substrate concentration yielded *k*_on_ and *k*_off_ of nucleotide exchange in table.

We monitored the interactions of ParA2 with MANT-labelled nucleotides using stopped-flow kinetics. The relative fluorescence increased upon MANT-ATP binding and the extent of binding increased with increasing ParA2 concentrations (Figure 3A). The binding curves took ∼30 s to reach an apparent steady state and fitted well to single exponential association with observed rates, *k*_obs_ 0.09–0.13 s^−1^ (Figure S3A). This timescale of binding ATP appears to be slow for a typical enzyme, suggesting that ParA2 may be undergoing dimerization or remodeling upon ATP-binding. However, at higher ParA2 concentrations, the MANT-ATP observed binding rate did not increase (Figure S3A). This suggests that ParA2 already exists as dimers at the lowest concentration tested (0.6 μM), prior to binding ATP. This is also consistent with the SEC-MALS data (Figure 2A). The pseudo-first-order rate constants, *k*_on_ and *k*_off_ were determined by titrations with increasing MANT-ATP concentrations (Figure 3B). The observed binding rates of MANT-ADP to ParA2 were slightly lower than MANT-ATP and did not increase with ParA2 concentrations (Figures 3C and S3A). *k*_on_ and *k*_off_ of MANT-ADP to ParA2 were also similar to those of MANT-ATP (Figure 3D), and their *K*_D_ (*k*_off_/*k*_on_) were both ∼8 μM, comparable to the steady state measurements (Figure S2). We examined the nucleotide dissociation rates by pre-incubating ParA2 with MANT-AXP (ATP or ADP) before mixing with unlabelled nucleotides. The subsequent decrease in MANT-ATP fluorescence was multiphasic, with an initial fast phase followed by a slower phase of *k*_obs_ 0.015–0.02 s^−1^ (Figures 3E and S3A), indicating that nucleotide release from ParA2 is slowed down by ATP hydrolysis. In comparison, MANT-ADP fluorescence dropped rapidly at *k*_obs_ 0.07–0.08 s^−1^, up to 5-fold faster than MANT-ATP dissociation (Figure S3A). In sum, both ATP and ADP bind ParA2 dimers with similar affinities, but ATP dissociation is slower due to ATP hydrolysis coupled with ADP release.

In growing cells, ParA2 dimers would undergo nucleotide exchange upon ATP hydrolysis. Depending on the rate of nucleotide exchange, this could limit the ATPase cycle. We investigated pseudo-first-order rate constants of nucleotide exchange rates by pre-incubating ParA2 with unlabeled nucleotide and rapidly mixing with increasing MANT-AXP concentrations (Figure 3F). *k*_on_ of ADP→ATP exchange (6.5×10^−4^ μM^−1^s^−1^) was similar to ADP→ADP exchange (6.1×10^−4^ μM^−1^s^−1^), although much slower than ATP→ATP exchange (1.4×10^−3^ μM^−1^s^−1^). In comparison, *k*_on_ values for nucleotide exchange (Figure 3F) were much lower than for nucleotide binding (Figure 3B, D). The slow rates for nucleotide exchange correspond to ADP release and subsequent MANT-AXP binding. The observed nucleotide exchange rates *k*_obs_ increased with increasing ParA2 concentrations, indicating a higher order dependence on protein concentrations (Figure S3B). Based on these data, ADP→ATP exchange appears to be a rate-limiting step in ParA2 ATPase cycle. This slow step in conversion of ParA2-ADP to ParA2-ATP dimers may contribute to the time delay in DNA rebinding, generating a ParA2 gradient on the nucleoid.

### ATP provides maximal stability to ParA2

**Figure 4.**
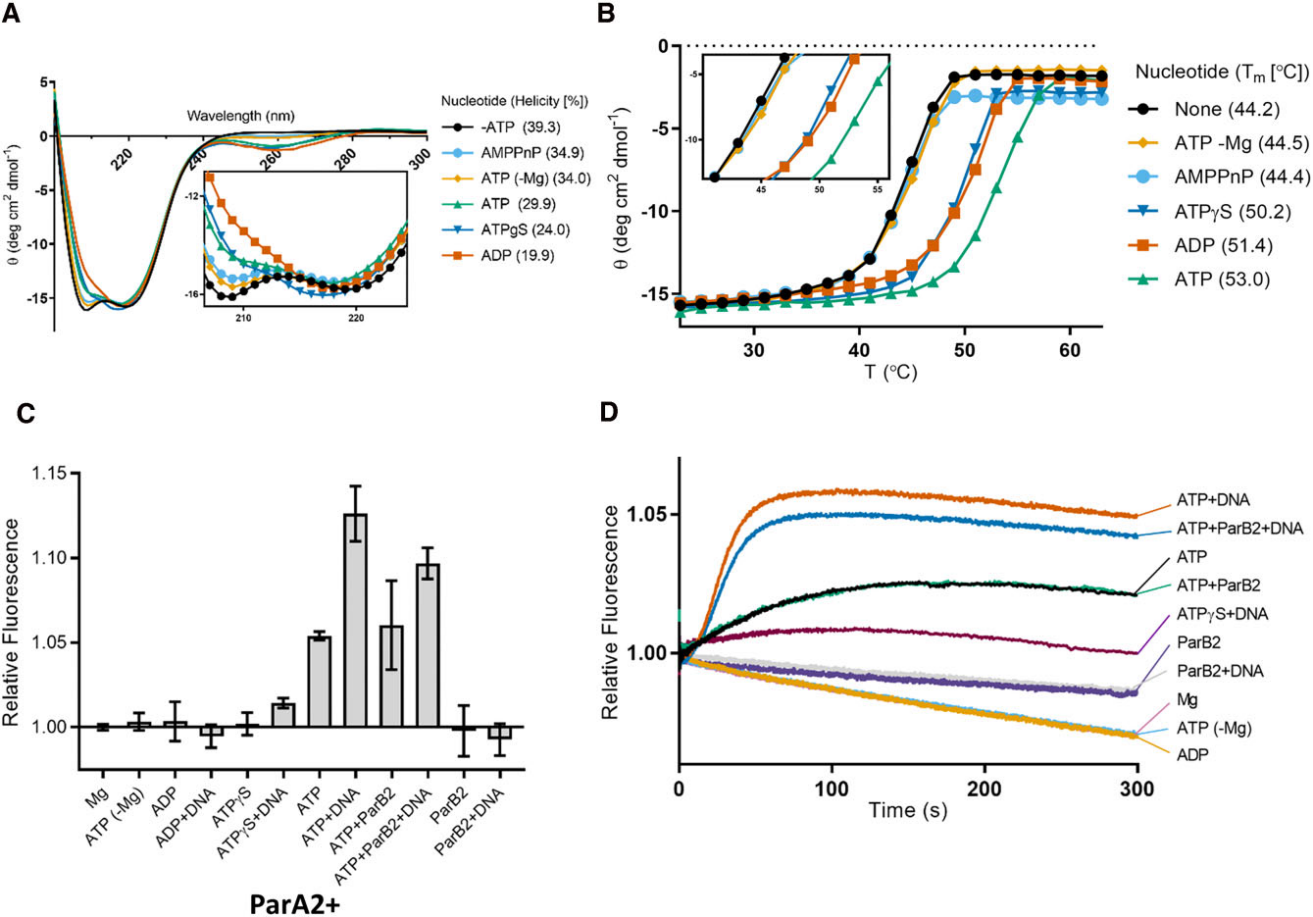
ParA2 conformational switch induced by adenine nucleotides and DNA. (**A**) CD spectra of ParA2 in presence of different adenosine nucleotides. The inset contains a zoomed-in section of θ from 206–224 nm. Surface helicity (%) in case of each nucleotide is indicated. 5 μM ParA2 was prepared in 10 mM Tris–HCl pH 8, 5 mM MgCl_2_ and 1 mM adenosine nucleotide when present. (**B**) The effects of adenosine nucleotides on ParA2 stability. ParA2 changes in secondary structure were monitored by CD at 220 nm (θ_220_), with an averaging time of 8 s, from 24°C to 63°C. Samples were prepared as in (A). Samples were equilibrated for 1 min prior to measurement with 2°C increments. The inset contains the zoomed-in section of *T*_m_ from 40–56°C. Relative ParA2 *T*_m_ values under different nucleotide conditions are also shown alongside the melting profiles. (**C**) Changes in steady state ParA2 tryptophan fluorescence. ParA2 (0.6 μM) incubated in Buffer B with nucleotide (1 mM) at 23°C for 400 s before relative fluorescence change was measured. In measurements without MgCl_2_, the buffer contained 0.1 mM EDTA instead of MgCl_2_. (**D**) Kinetics of ParA2 tryptophan fluorescence change. 0.6 μM ParA2 in Buffer B at 2× final concentration in syringe 1 was rapidly mixed with 1 mM nucleotide, 0.1 mg ml^−1^ DNA and 0.6 μM ParB2 (where indicated) in Buffer B at 2× final concentration at 23°C in syringe 2 of the stopped-flow apparatus.

As nucleotide exchange occurs at a slow rate, we wanted to investigate if nucleotide binding induces a conformational change in ParA2 dimers. Effects of various adenine nucleotides on ParA2 secondary structure and conformational stability were tested using circular dichroism (CD) spectroscopy. The presence of adenine nucleotides shifted the CD spectra considerably toward higher molar ellipticity (θ) at 208 nm and slightly at 220 nm, indicating a decrease in proportion of α-helices (Figure 4A). Concurrently, a slight decrease at 218 nm showed an increase of β-sheets. The overall spectra resulted in a decrease of ParA2 helicity by 10% with ATP and by 20% with ATPγS and ADP, indicating conformational changes of ParA2 dimers upon nucleotide interactions. A similar spectral shift and loss of helicity was also observed for F SopA, suggesting analogous conformational changes upon nucleotide-binding (58). In contrast, P1 ParA showed a slight increase in helicity (4–5%) upon ADP binding (2,7). This was corroborated by recent crystal structures of apo and ParA2-ADP dimers showing slight shifts in the P-loop and the domain-swapping region (47). The effect of adenine nucleotides on ParA2 structural stability was examined by thermal melts and the molar ellipticity (θ) monitored at 220 nm between 23°C and 63°C (Figure 4B). The denaturation of ParA2 occurred at *T*_m_ = 44°C and was irreversible as ParA2 precipitated at the end of the heating cycle. ATP stabilized ParA2 structure at most by 20%, conferring a maximal *T*_m_ = 53°C. Both ATPγS and ADP raised *T*_m_ to 50°C and 51°C, respectively. AMPPNP or ATP in the absence of Mg^2+^ had no effect on ParA2 stability. A similar effect of ATP and ADP on *T*_m_ was also observed for P1 ParA and F SopA (58,59), indicating that this may be a common feature among chromosome and plasmid ParAs, where nucleotide-binding stabilizes ParA conformation in general although the extent of stabilization may vary depending on the ParA in question.

### ParA2 undergoes slow remodeling to ParA2*-ATP dimer

To further investigate ParA2 structural changes upon interactions with cofactors- adenine nucleotides, DNA or ParB2, we measured intrinsic tryptophan fluorescence of the protein. ParA2 monomer (407 aa), has six tryptophan residues at positions 111, 224, 267, 287, 308 and 401. Sequence alignment with P1 ParA shows that residue W224 of ParA2 corresponds to W216 of P1 ParA, located on the α-helix 11 and close to the P1 ParA-ADP dimer interface (31,60). This indicates that intrinsic fluorescence could be used to monitor conformational changes of ParA2, as was the case with P1 ParA (31). Negative controls without ATP or Mg^2+^ showed no change in tryptophan fluorescence (Figure 4C, bars 1, 2), while the presence of ATP and Mg^2+^ increased fluorescence by 5% (Figure 4C, bar 7). ADP and ATPγS had little effect on the fluorescence change, indicating that ParA2 remodeling is ATP + Mg^2+^ specific (Figure 4C, bars 3, 5). Addition of DNA with ATP increased fluorescence by 13%, implying further remodeling when ParA2-ATP dimers bind DNA (Figure 4C, bar 8). Although ATPγS did not initially cause a change in ParA2 tryptophan fluorescence, DNA was able to effect a slight increase, suggesting that DNA induces a ParA2 conformation that is different than with ATP (Figure 4C, bar 6). As ParB2 interacts directly with ParA2 to stimulate ATP hydrolysis (Figure 2B, C), we wanted to investigate if interactions with ParB2 has an effect on ParA2 structure. ParB2 (323 aa) has two tryptophan residues at positions 246 and 268 but negative controls showed negligible fluorescence change with ATP and DNA. This demonstrates that ParB2 could be used as a cofactor in this assay without interfering with ParA2 tryptophan fluorescence (Figure 4C, bars 11, 12). Interestingly, we found that the fluorescence change of ParA2 with ATP remained similar even in the presence of ParB2 (Figure 4C, bar 9). The fluorescence increase with DNA was slightly dampened by ParB2, suggesting a separate conformation of ParA2 DNA complex when interacting with ParB2 (Figure 4C, bar 10).

To investigate the kinetics of ParA2 conformational change between different cofactors, we monitored ParA2 tryptophan fluorescence change using stopped flow. Consistent with the steady state measurements, the negative controls of ParA2 without ATP or Mg^2+^ showed a prolonged decrease in fluorescence that is attributed to photobleaching (Figure 4D). Similarly, the lack of fluorescence change by ADP and ATPγS indicates that the nucleotides did not induce any noticeable conformational change despite binding to ParA2. On the other hand, ParA2 with ATP showed a surprisingly slow hyperbolic increase to reach an apparent steady state in ∼180 s (Figure 4D). The curves fitted well to single-exponential with similar rates of *k*_obs_ 0.015–0.018 s^−1^ and were not dependent on ParA2 concentrations. (Figure S4A, S4E). The rates of conformational change were slower than MANT-ATP binding by at least 6-fold (Figure S3A) and nucleotide exchange by up to 3-fold at higher ParA2 concentrations (Figure S3B). We hypothesize that this fluorescence change is due to a slow remodeling of ATP-bound ParA2 dimers to a distinct intermediate state, ParA2*_2_-ATP_2_, analogous with P1 ParA. As ATP binding and nucleotide exchange have faster observed rates, we infer the slow remodeling to be the rate-limiting-step in the ATPase cycle. We predict that the presence of DNA will catalytically induce structural rearrangement of ParA2 to the ParA2*_2_-ATP_2_ intermediate state that is active for DNA binding.

### DNA modulates kinetics of ParA2-ATP remodeling

We tested this hypothesis by rapidly mixing nonspecific DNA and ATP with ParA2. Tryptophan fluorescence of ParA2 showed an initial lag phase before rising sharply to reach steady state at ∼80 s (Figure 4D). The peak fluorescence increased to more than 2-fold in intensity and reached at 2x faster rate than those for ParA2 with ATP alone. The initial lag phase was most likely due to nucleotide-binding step preceding the remodeling of ParA2-ATP dimers. Hence, the intensity curve was fitted with a single exponential excluding the initial lag phase. Strikingly, compared to ParA2 with ATP alone, the presence of DNA sped up *k*_obs_ to 0.03–0.07 s^−1^, increasing toward higher ParA2 concentrations (Figure S4E). This dependence on ParA2 concentration suggest higher-order protein interactions with DNA that induce cooperative remodeling of ParA2–ATP dimers to the DNA-bound state (Figures 4D, S4C, S4E). When we mixed ParA2 with ATPγS, we found that ParA2 showed a slight fluorescence increase only with DNA, indicating that DNA induces ParA2 remodeling that is not dependent on ATP hydrolysis. We believe that the recent structure of DNA-bound ParA2–ATPγS dimers reflects that of the ATP-bound intermediate, where ParA2 dimers undergoes structural rearrangement in the cross-dimer interaction to cooperatively oligomerize on DNA (47). Altogether, these data imply that DNA activates the rate of conformational change of ParA2–ATP dimer, and lowers the energy barrier toward the ParA2*–ATP dimer intermediate that is primed for DNA binding. ParA2 conformational change reaches steady state about 5-fold faster compared to P1 ParA, indicating that ParA2 is able to switch more quickly from a non-binding state to an active DNA-binding state. We suggest that this key feature distinguishes chromosomal from plasmid ParAs, enabling ParA2 to cooperatively rebind DNA more quickly and to be more dynamic in exploiting and patterning the nucleoid as a scaffold for Chr2 segregation.

When we investigated the effect of ParB2 on the kinetics of ParA2 tryptophan fluorescence, we found that surprisingly ParB2 did not change the rates of fluorescence increase, with or without DNA (Figures 4D, S4B-E). From these data, we infer that DNA is the primary cofactor in modulating ParA2 conformational switch since ParA2-ParB2 interactions did not appear to change the conformational kinetics or extent of ParA2*-ATP dimer formation. We postulate that ParB2 interacts with ParA2 by inserting an arginine finger or α-helix at the ParA2 dimer interface to stimulate ATP-hydrolysis (56,61,62). Based on the overlay of TP228 ParA–ParB (5U1G) and pNOB8 ParA–DNA (5U1J) crystal structures, the location of ParB N-terminal helix at the ParA dimer interface clashes with the DNA-binding region (61). Thus, it was reported that ParB stabilizes ParA nucleotide sandwich dimer but not the DNA-binding state. However, when we overlay TP228 ParA–ParB (5U1G) (61) with *Vc* ParA2–ADP crystal structures (7NPE) and ParA2–ATPγS–DNA cryo-EM structures (7NPF) (47), we found that the ParB helices do not clash with ParA DNA-binding regions (Figure S4F). Instead, we infer that although ParB2 does not alter the kinetics or extent of ParA2 remodeling, ParB2 helices can interact with ParA2–DNA complexes at the dimer interface and stimulate ATP hydrolysis without causing further conformational changes.

### ParA2*-ATP dimers bind DNA cooperatively

**Figure 5.**
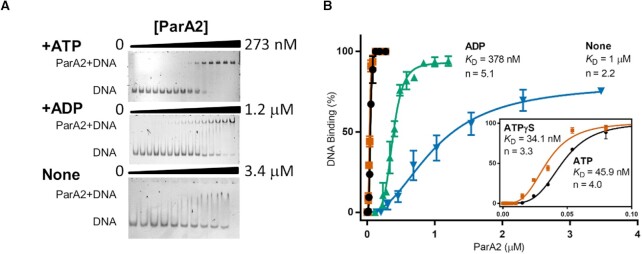
ParA2 binds DNA cooperatively in the presence of ATP. (**A**) EMSA of ParA2 binding to Cy3-69 bp non-specific DNA (5 nM). ParA2 at increasing concentrations as indicated was titrated with 5 nM Cy3-labeled 69 bp DNA in Buffer A, in the presence of 2 mM ATP (top), ADP (centre) or without nucleotides (bottom). The samples were run in a 5% polyacrylamide gel in TBM buffer. The positions of free DNA and the ParA2–DNA complexes are indicated on the left. (**B**) ParA2–DNA binding affinity. % DNA bound was calculated using ImageJ. Data was plotted, and the derived *K*_D_ values and Hill coefficients (*n*) under each condition are stated.

In general, ParAs bind DNA nonspecifically, coating the nucleoid to mediate plasmid or chromosome segregation. ParA2 forms various structured helical filaments on DNA that is nucleotide-dependent (46,47). To evaluate ParA2 binding properties on DNA, we performed EMSAs using Cy3-labeled 69 bp non-specific DNA and adenine nucleotides. A sharp retarded band of ParA2-DNA complexes was seen with increasing intensities at higher ParA2 concentrations (Figure 5A). The binding curves showed that ParA2 had similar affinities with ATP (46 nM) and ATPγS (34 nM), demonstrating that ATP hydrolysis is not required for DNA binding (Figure 5B) or conformational change (Figure 4D). The binding curves were sigmoidal, showing that ParA2 binds DNA cooperatively in the presence of ATP with a Hill coefficient, *n* = 4. This implies that two dimers of ParA2*-ATP bind to 69 bp DNA (∼30 bp/dimer) and suggests that ParA2 has a propensity to form higher-order complexes on DNA via dimer-dimer interactions. With ADP, ParA2 could not achieve full binding even at 1.2 μM and had 8-fold lower affinity for DNA (378 nM). Without nucleotide, the bands were smeared throughout the lanes, showing that ParA2 by itself binds DNA too weakly (1 μM) to be stable during electrophoresis (Figure 5A, B). To test for ParA2 dissociation from DNA, unlabeled sonicated salmon sperm DNA (sssDNA) was added to preformed ATP-bound ParA2-DNA complexes (Figure S6A, C). sssDNA facilitated disassembly of the complex in a concentration dependent manner. This was also observed with ATPγS, indicating that ParA2 is able to dissociate from DNA without ATP hydrolysis. Together, the results show that upon ATP binding, ParA2 dimer undergoes slow remodeling to ParA2*–ATP dimer, a DNA-binding competent state. DNA catalyzes this slow step and licenses ParA2 to cooperatively bind onto DNA to form higher order [ParA2*_2_–ATP_2_]_*n*_ oligomers to dynamically coat the nucleoid.

### *In vivo* dynamics and *in vitro* interactions of K124 variants of ParA2 with ATP and DNA

**Figure 6.**
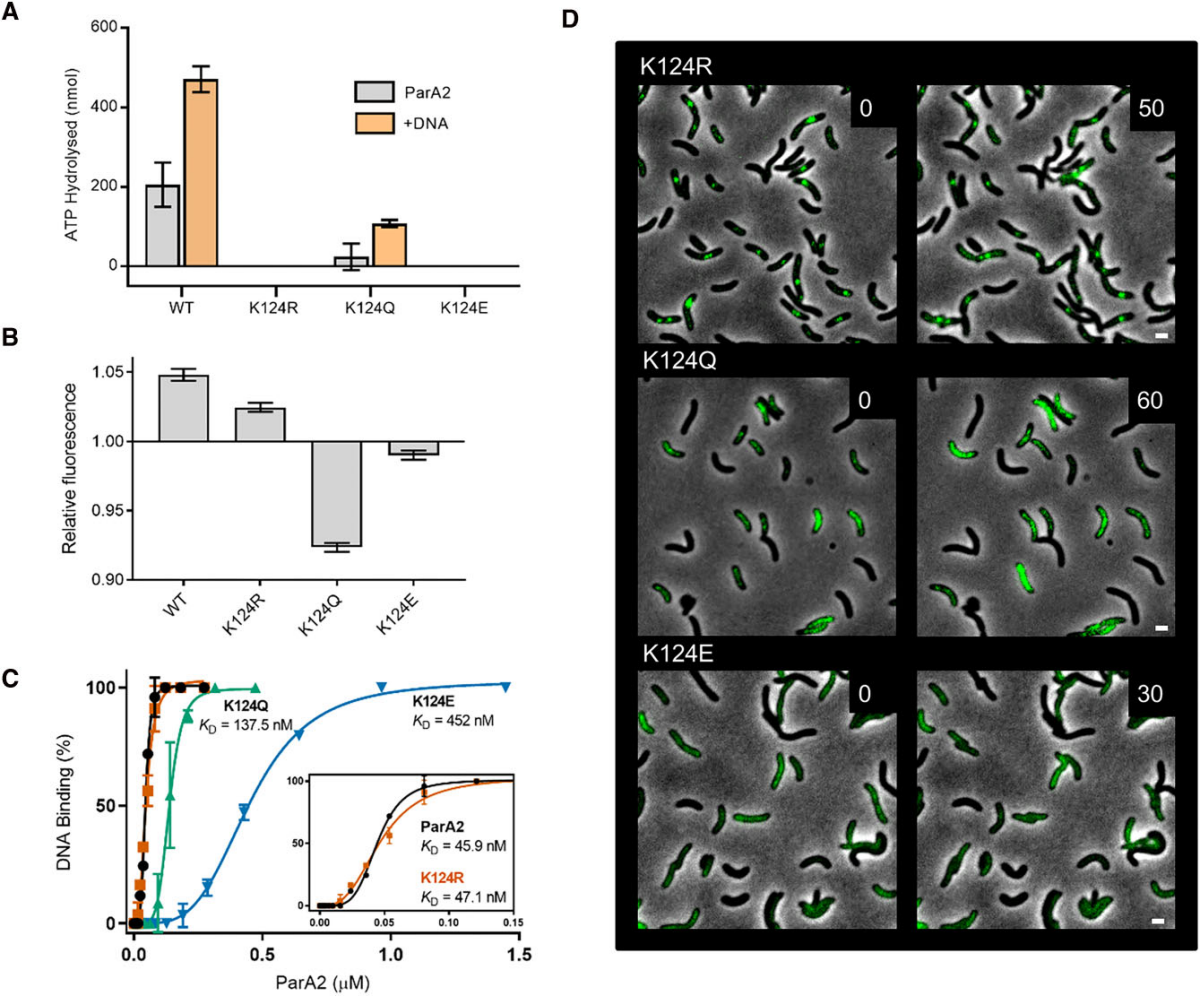
Interactions of ParA2 K124 variants with ATP and DNA, and localization of the variants in *V. cholerae* cells. (**A**) ATP hydrolysis of ParA2 K124 variants. 1.5 μM ParA2 WT or its variants K124R, K124Q and K124E was mixed in Buffer A with 200 μM ATP spiked with 64 nM [α-32P]-ATP. 100 μg ml^−1^ sonicated salmon sperm DNA was added as indicated. The hydrolysis product was measured after 40 min incubation at 23°C. (**B**) Extent of MANT-ATP binding of ParA2 variants. 1.5 μM WT ParA2 or its indicated variants was mixed with MANT-ATP in Buffer B on ice. Relative fluorescence change at steady state was measured at 0 min and after incubating at 37°C for 20 min. (**C**) Comparison of DNA-binding activity of WT ParA2 with its variants by EMSA. The proteins were incubated with Cy3-69 bp DNA (5 nM) in the presence of 2 mM ATP and the mixtures were run in a 5% polyacrylamide gel. DNA-binding (%) was calculated using ImageJ. Data were plotted and fitted to determine *K*_D_ values. (**D**) *In vivo* imaging of GFP-fused to ParA2 K124 variants. The proteins were expressed in *V. cholerae* cells and are not found to show dynamic localizations. Numbers are in min and scale bars represent 2 μm.

We further examined the roles of ATP binding and hydrolysis in ParA2 function and their effects on *in vivo* dynamics. Three ParA2 mutants were constructed by substituting a conserved lysine residue at 124 in the Walker A box with glutamine (K124Q, uncharged side chain), glutamic acid (K124E, negatively charged side chain), or arginine (K124R, positively charged side chain). These are analogous to the P-loop P1 ParA mutants that showed defective ATPase activity (63). All purified ParA2 mutant proteins showed defects in ATP hydrolysis (Figure 6A). K124Q was the only mutant that maintained some ATPase activity and reduced stimulation by DNA compared to WT ParA2. K124R and K124E displayed no ATPase activity and no stimulation by DNA. End-point fluorescence measurements showed that K124R bound MANT-ATP to a lower extent than WT ParA2 (Figure 6B). K124Q showed fluorescence quenching, suggesting interaction with MANT-ATP. K124E had negligible MANT-ATP fluorescence change, indicating lack of ATP binding. DNA-binding activities of the mutants were determined by EMSA using a Cy3-labeled 69 bp nsDNA fragment and ATP (Figure 6C). K124R had a similar affinity for DNA as WT ParA2 (*K*_D_ = 47.1 nM), despite being deficient in ATP hydrolysis. K124Q had a 3-fold decrease in DNA affinity (*K*_D_ = 137.5 nM) that is attributed to aberrant ATP-binding. K124E had the lowest DNA affinity (*K*_D_ = 452 nM) due to deficient ATP binding. EMSAs of K124R and K124Q nucleoprotein complexes showed more resistance to dissociation, while K124E complexes dissociated completely at lower competitor DNA concentrations (Figure S6B). This corroborates the cryo-EM data where K124R and K124Q but not K124E, formed filaments on DNA with ATP (47). These findings indicate that binding to and dissociation from DNA are not dependent on ParA2 ATPase activity. ParA2–ATP is thus able to exchange on DNA without hydrolysing ATP.

To investigate the effects of K124 substitutions on ParA2 *in vivo*, we expressed C-terminally GFP-fused K124 variants from plasmids in the presence of WT ParA2 in *V. cholerae* cells and performed time-lapse imaging as before (Figure 6D). Most of the cells appeared to grow and divide, albeit slower than cells expressing ParA2-GFP, indicating that transient expression of the mutants are not grossly toxic to the cells. Intriguingly, K124R-GFP formed punctate foci at the mid or quarter cell positions that remained immobile over time (Figure 6D, top). We infer that K124R-GFP binds to the ParB2-*parS2* complexes but fails to turnover and dissociate from the partition complexes, sequestering K124R-GFP from patterning the surrounding chromosomal DNA and preventing *ori2* segregation. This is analogous to the P1 *par*^PD^ (propagation defective) mutant ParA[K122R] that irreversibly anchors reconstituted partition complexes to the DNA carpet and blocks complex dynamics (32). K124Q-GFP was localized throughout the cells (Figure 6D, middle). We did not observe any dynamic oscillations as with ParA2-GFP. However, in some cells, K124Q-GFP appeared as patches and seemed to ‘flicker’, suggesting some binding and dissociation from the nucleoid. This demonstrates that the residual ATPase activity of K124Q is insufficient to produce consistent dynamic oscillations in cells. K124E-GFP appeared diffused and more evenly distributed throughout the cells (Figure 6D, bottom). Again, no protein oscillations was observed. Together with the biochemistry data these results highlight the importance of the ATPase activity of ParA2 for its dynamic oscillations.

### ATP stimulates ParA2*-GFP binding and dissociation on DNA carpet

**Figure 7.**
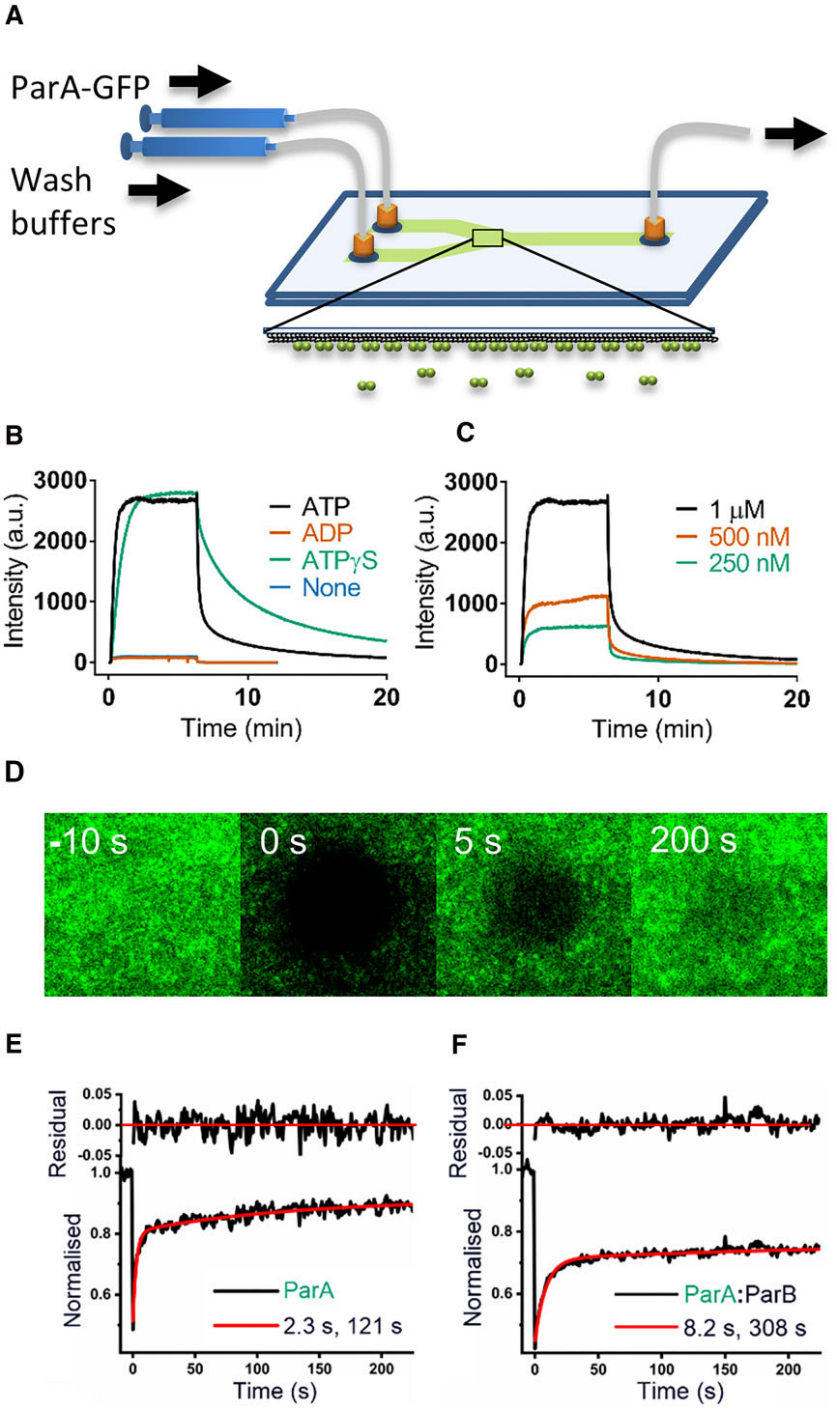
Exchange of ParA2 on DNA carpet and its dampening by ParB2. (**A**) Infusion of ParA2-GFP into a 2-inlet flowcell coated with DNA carpet and disassembly of ParA2-GFP from the carpet by wash buffer. A small spot was imaged at the Y-junction with TIRF microscope for measuring ParA2-GFP binding and dissociation. (**B**) Binding and dissociation curves of ParA2-GFP on DNA carpet. ParA2-GFP (10 μM) was preincubated in the presence or absence of ATP, ADP or ATPγS (2 mM). Protein was diluted to 1 μM before infusion into DNA-carpeted flowcell and at 6 min after infusion when steady state has been achieved, the flow was switched to wash buffer. (**C**) ParA2 binds DNA carpet cooperatively at higher protein concentrations. ParA2-GFP (10 μM) was preincubated with ATP (1 mM) prior to dilution to final concentrations shown. Samples were infused into flowcell and washed as in (A). All binding and dissociation rates are listed in Table S1. (**D**) Time-lapse images of ParA2-GFP (low density) recovery on DNA carpet after photobleaching at 0 s. (**E**) Representative FRAP curve (black line) of ParA2-GFP was fitted to a double-exponential (red line). The fitted time constants correspond to a fast species (2.3 s) and slow species (121 s). (**F**) Representative FRAP curve (black line) of ParA2-GFP:ParB2 mixed at 1:2 ratio was fitted to a double exponential (red line). The fitted time constants correspond to a fast species (8.2 s) and slow species (308 s). All FRAP data are listed in Table S2.

To visualize ParA2 interactions on DNA, we constructed and purified ParA2 with C-terminally fused GFP. ParA2-GFP was only mildly toxic *in vivo* and its functionality *in vitro* was equivalent to biochemical properties as WT ParA2 (Figure S5). We coated a two-inlet flowcell surface with a DNA carpet acting as a biomimetic chromosome and imaged ParA2-GFP with a TIRF microscope (Figure 7A). The two-inlet flowcell allows us to instantly switch between flowing sample solution and wash buffer to monitor real-time binding and dissociation kinetics of ParA2-GFP on the DNA carpet (see Materials and Methods). ParA2-GFP was shown by EMSA to have similar DNA binding activity compared to that of WT ParA2 (Figure S6). We pre-incubated 10 μM ParA2-GFP with 2 mM of various adenine nucleotides and diluted the mixture 10-fold (to 1 μM ParA2-GFP) before infusing into the flowcell. This helps to saturate nucleotide-binding to ParA2 dimers so that the observed kinetic changes would be attributed primarily to DNA-binding of dimers. Upon infusing with ATP, ParA2-GFP bound to the DNA carpet instantly and exponentially at rate of 0.05 s^−1^ and reached steady state within 1 min (Figure 7B, Table S1). When we switched to wash buffer at 6 min, ParA2-GFP intensity dropped immediately, dissociating at twice the rate of binding (0.1 s^−1^). ParA2-GFP coated the DNA carpet thoroughly and uniformly, and dissociated evenly with wash buffer, indicative of highly efficient reversible binding (Movie S2). In the presence of ATPγS, although ParA2-GFP bound the DNA carpet to a similar extent as with ATP, the binding and dissociation rates were both slower (∼0.02 s^−1^, Table S1). This supports our EMSA data that ATP hydrolysis is not required for DNA interactions but does stimulate ParA2-DNA binding (3-fold) and dissociation (7-fold). When infused with ADP or without nucleotides, ParA2-GFP showed negligible binding to DNA carpet, contrary to a previous study using negative staining (46). We believe that the apparent low affinity binding in the flowcell is due to high concentration ratio of nonspecific DNA to protein, a condition likely to mimic intracellular environment more closely.

Increasing ParA2-GFP concentrations doubled the DNA binding from 250 to 500 nM but showed higher order concentration dependence at 1 μM, suggesting cooperative binding (Figure 7C). All three concentrations had a similar binding rate of 0.05 s^−1^ and dissociation rates of 0.11–0.19 s^−1^ (Table S1). These dissociation rates are 5–8-fold faster than those obtained with P1 ParA (0.8–1.8 min^−1^) and F Sop A (1.9 min^−1^) (31,32). The dissociation rates slightly increased in the presence of ParB2 and DNA in the wash buffer, but whether this slight differences are significant remain to be established. If the differences prove to be real, they could be due to stimulated ATP hydrolysis and DNA dissociation by the cofactors (Figure S7).

From our live-cell imaging of *V. cholerae*, ParA2-GFP took 30–60 s to dissociate from the nucleoid and transit across the cell (Figure 1). These fast dynamic oscillations of ParA2-GFP observed in the cell corroborate well with the rapid dissociation rates measured on DNA carpet that is ATP-stimulated. In contrast, when we mixed ParA2-GFP and ATP just before infusing into the flowcell in an ‘ATP-start’ experiment, there was an initial lag time of 1 min before intensity increased (Figure S8). We attribute this lag to ATP-binding. Notably, the extent of binding dropped 5–10-fold and the binding rates were slower by 3-fold compared to pre-incubation experiments. These results support our tryptophan fluorescence data, showing a time delay switch upon ATP-binding for ParA2-GFP to transition to an active DNA-binding state.

### Rapid exchange of ParA2*-GFP on DNA carpet is stabilized by ParB2

To characterize the mobility of ParA2-GFP on DNA carpet, we performed fluorescence recovery after photobleaching (FRAP). If ParA2 forms extended stable filaments on DNA, we would expect a large fraction of unrecovered to slowly recovered species on the DNA carpet depending on protein turnover. If instead there is fast recovery of ParA2-GFP on the carpet, this indicates protein exchange on DNA, where ParA2 dimers unbind and rebind DNA from solution phase. ParA2-GFP was infused into the flowcell at two different densities (28% and 100% at steady state), which are in excess of estimated physiological density of ∼1% (32). A spot was photobleached for < 1 s on the ParA2-GFP-coated carpet and the fluorescence recovery monitored (Figure 7D, Movie S3). At 28% density, we found only a small fraction of immobile species (13%). Majority of ParA2-GFP bleached spots belonged to a fast species and recovered rapidly at 0.43 s^−1^ (64%, Figure 7E and Table S2). As most of the proteins were DNA-bound and recovered by exchanging with ParA2-GFP from solution phase, the photobleaching recovery rate is expected to be dependent on the rate of protein unbinding. At 100% density or steady state binding, ParA2-GFP recovery rate decreased to 0.12 s^−1^ and a fraction of fast species shifted towards slower and immobile species (Movie S4 and Table S2). This bimodality in dissociation suggests that with increasing protein to DNA ratio, there is an increasing population of ParA2-GFP that is forming higher-order complexes with conformational changes on DNA that is exchanging at a slower rate. The exchange rate is also consistent with the dissociation rates determined from the wash experiments (Table S1). When compared to plasmid ParAs, the rate of recovery of ParA2 was 15- and 6-fold faster than that of P1 ParA (1.8 min^−1^) and F Sop A (4.7 min^−1^), respectively (32,33). These data show that the exchange rate of ParA2 on DNA is clearly faster compared to plasmid ParAs. Here, we did not observe any indication of stable ParA2-ATP filaments on DNA. However, it is likely that higher protein densities lead to cooperative binding of ParA2 to form higher order oligomers or filaments on DNA (46,47).

As ParB2 stimulates ParA2 ATPase activity, we wanted to test if ParB2 also affects ParA2 exchange on DNA. We found that ParB2 lowers ParA2-GFP density on DNA carpet and inhibits protein exchange, increasing recovery time by up to 4-fold at higher ParB2 to ParA2 ratio (Figure 7F, Table S2). Furthermore, the major fraction of faster species was reduced in the presence of ParB2, converting it to the immobile fraction. This indicates that ParB2 stabilizes ParA2 binding on DNA and slows down its unbinding. A similar effect was also reported for P1 ParA and F SopA (32,33). These results imply the presence of two separate populations of ParA: one that is DNA-bound and another that is interacting with ParB on the partition complex. We infer that ParB2, when bound to the partition complex at high concentrations, slows down ParA2 exchange on the partition complex and its vicinity, allowing for longer-lived depletion of ParA2 from DNA. Collectively, our data – fast ParA2-DNA exchange and the absence of stable filaments on DNA carpet—contradict the filament model where ParA2 forms extended polymers on DNA that is stable enough to exert a pulling force on the chromosome. Instead, our data support a diffusion-ratchet based mechanism where ParA2 cooperatively binds Chr2 as dynamic oligomers to pattern the nucleoid, mediating its movement and bidirectional segregation.

## DISCUSSION

### Kinetics of ParA2 ATPase cycle and DNA rebinding

**Figure 8.**
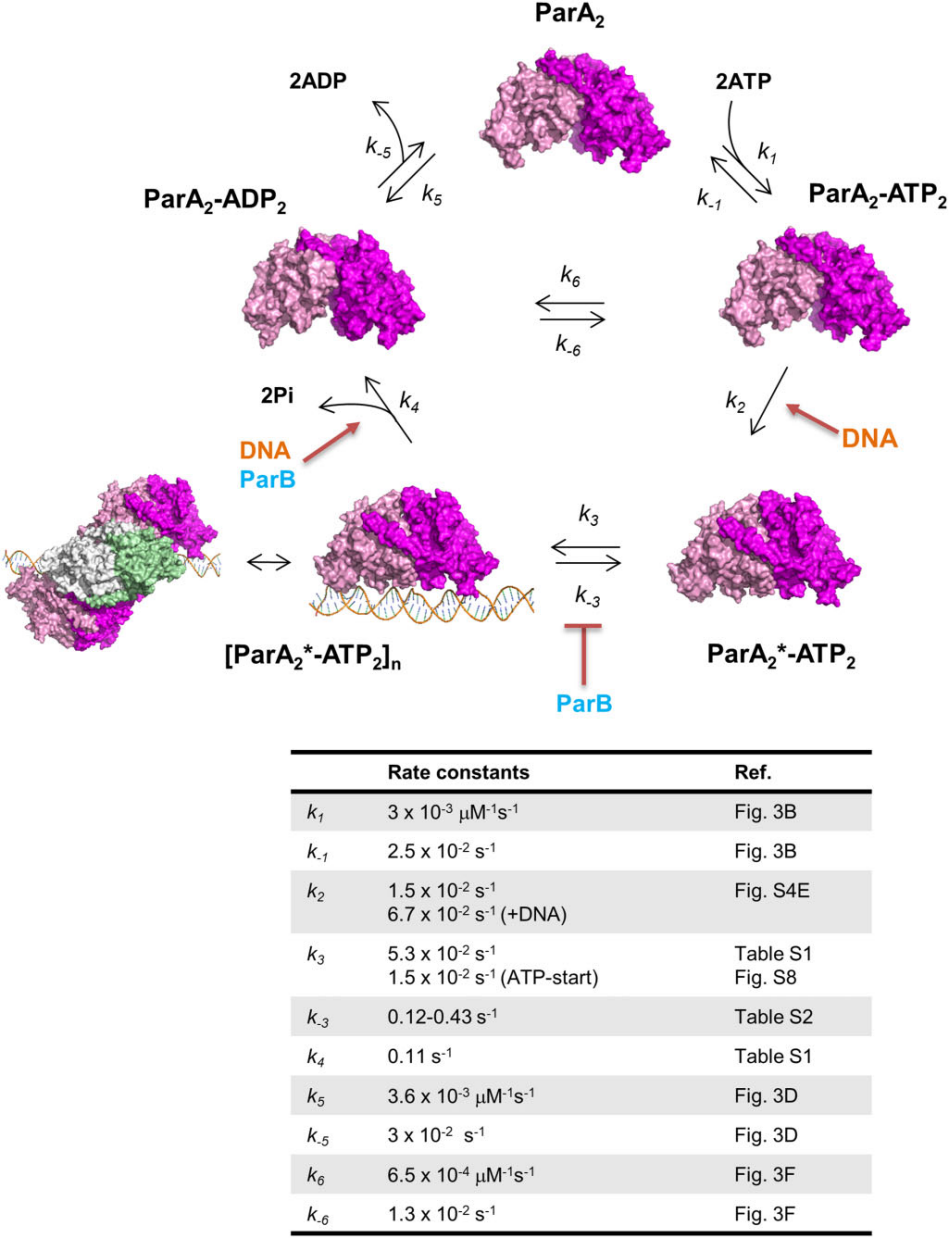
Kinetic pathway and rates for ATP control of ParA2-DNA interactions. ParA2 dimers (magenta and pink) bind ATP to form a closed sandwich dimer (*k*_1_, *k*_–1_). ParA2_2_-ATP_2_ undergoes a slow remodeling to active ParA2_2_*–ATP_2_ state (*k*_2_), an open dimer that licenses ParA2 dimers to bind DNA. This slow transition is accelerated in the presence of DNA. Once competent for DNA-binding, ParA2_2_*–ATP_2_ loads onto DNA (orange) and induces cooperative binding of more ParA2* dimers via dimer-dimer interactions to form oligomers on DNA (*k*_3_, *k*_–3_). Orthogonal ParA2 dimers are coloured green and white for clarity. ParB2 inhibits ParA2* exchange on DNA as it stabilizes ParA2*–DNA interactions. ParA2_2_–ADP_2_ dissociates from DNA (*k_4_*) upon ATP hydrolysis that is stimulated by DNA and ParB2. Once ADP dissociates from ParA2 dimers (*k*_5_, *k*_–5_), the cycle restarts and ParA2 dimers diffuse away and are ready to rebind ATP for the next round of DNA-binding. ParA2 dimers undergo nucleotide exchange (*k*_6_, *k*_–6_) at much slower rates than ATP-binding. ParA2 recruitment on DNA and its dynamic oscillations are primarily controlled by the rate-limiting conformational switch to its active state, and the secondary control by the nucleotide exchange rate. (*k*_2_ and *k*_3_, *k*_4_are rates at 1.25 μM and 1 μM ParA2, respectively.) [ParA2 apo and ParA2-ADP X-ray structures are from PDB:7NPD and 7NPE, respectively; the ParA-ATPγS-DNA cryo-EM structure is from PDB:7NPF (47)]

Here, we report the rate constants of ATPase cycle and DNA binding of a chromosomal ParA that mediates segregation of *V. cholerae* Chr2 (Figure 8). ParA2 forms spontaneous dimers at < 0.6 μM. Upon binding ATP (*k*_1_, *k*_–1_), ParA2_2_-ATP_2_ undergoes slow remodeling to ParA2_2_*–ATP_2_ state (*k*_2_) that licenses the dimers to bind DNA. This transition to the DNA binding state is accelerated in the presence of DNA by 2- to 5-fold. Once competent for DNA-binding, ParA2_2_*–ATP_2_ loads onto DNA and induces cooperative binding of more ParA2 dimers via dimer-dimer interactions to form higher-order oligomers on DNA, [ParA2_2_*–ATP_2_]_*n*_ (*k*_3_, *k*_–3_). Although, ParB2 did not appear to influence ParA2 conformational change, ParB2 inhibited ParA2 exchange by stabilizing ParA2-DNA interactions. High local concentrations of ParB2 on the partition complex triggers ParA2 dissociation by stimulating ATP hydrolysis. Upon hydrolysis and release of two inorganic phosphates, ParA2_2_–ADP_2_ dissociates from DNA and diffuse away (*k*_4_). Once ADP dissociates from ParA2 dimers (*k*_5_, *k*_–5_), the dimers are ready to rebind available ATP for the next round of DNA binding and the cycle restarts.

ParA2 dimers undergo nucleotide exchange (*k*_6_, *k*_–6_) at much slower rates than ATP binding (*k*_1_, *k*_–1_). Although the rate of ParA2 rebinding DNA is primarily determined by the rate-limiting conformational switch to the active state, there is also a secondary dependence on the nucleotide exchange rate. Particularly, once DNA activates ParA2 remodeling, slow nucleotide exchange regulates DNA rebinding and dynamic localization. This allows more time for ParA2 to diffuse and redistribute in the cell before rebinding the nucleoid. This delay in ATP recovery inhibits ParA2 from rebinding to the same location from where it dissociated and promotes ParA2 redistribution to the opposite pole. We predict that lower nucleotide exchange rate, lead to slower DNA rebinding, generating faster ParA2 oscillations. In MinCDE system, it has been shown that nucleotide exchange rates regulate the spatial distribution of MinD rebinding and oscillations (64). Thus, it is likely that ParA2 dynamic patterns on the nucleoid are spatiotemporally regulated by ParA2 remodeling as well as nucleotide exchange.

### Comparison of *Vc* ParA2 with ParA homologs

Our studies revealed many similarities but also significant differences in the activities of ParA2 with other plasmid and chromosomal ParAs. Unlike the ATP-dependent dimerization of plasmid ParAs (P1, F, TP228), *C. crescentus* ParA, *T. thermophilus*, *B. subtillis* and *H. pylori* Soj, *Vc* ParA2 forms dimers spontaneously without nucleotide. *Vc* ParA2 has higher ATP and ADP binding affinities (*K*_D_ ∼8 μM) compared to other ParA homologs by almost an order of magnitude: P1 ParA 30 μM (ATP) and 50 μM (ADP) (31,59), F SopA 74 μM (ATP) (30), *C. crescentus* ParA 50–60 μM (ATP) (65) and TP228 ParF 100 μM (ATP) (66). Significantly, *Vc* ParA2 shows higher *k*_cat_ than other plasmid and chromosomal ParAs, with greater ATP turnover rate (Figure S1B, S1C) (17,54–56,65,67–69). The stimulatory effects by ParB2 and DNA are also more pronounced (except *Bs* Soj). Despite ParA2 and P1 ParA sharing the same rate-limiting step, ParA2 remodeling is faster, leading to faster DNA-rebinding. We infer that in the cell, the fast exchange rates of ParA2 on the nucleoid will lead to fast on and off rates with its cognate partner ParB2. These faster transient interactions between ParA2 and the nucleoid, as well as ParA2 and ParB2-*parS2*, allow for the chromosome partition complex to be more dynamic in interacting with the nucleoid, relative to plasmids. Overall, the faster reaction rates compared to other ParA homologs imply that *Vc* ParA2 is a more efficient and robust enzyme, and selection of its dynamic features may have been necessitated to efficiently segregate a larger chromosomal cargo in coordination with Chr1 segregation and the cell cycle.

### Tug-of-war model of Chr2 segregation driven by oscillating ParA2 waves

**Figure 9.**
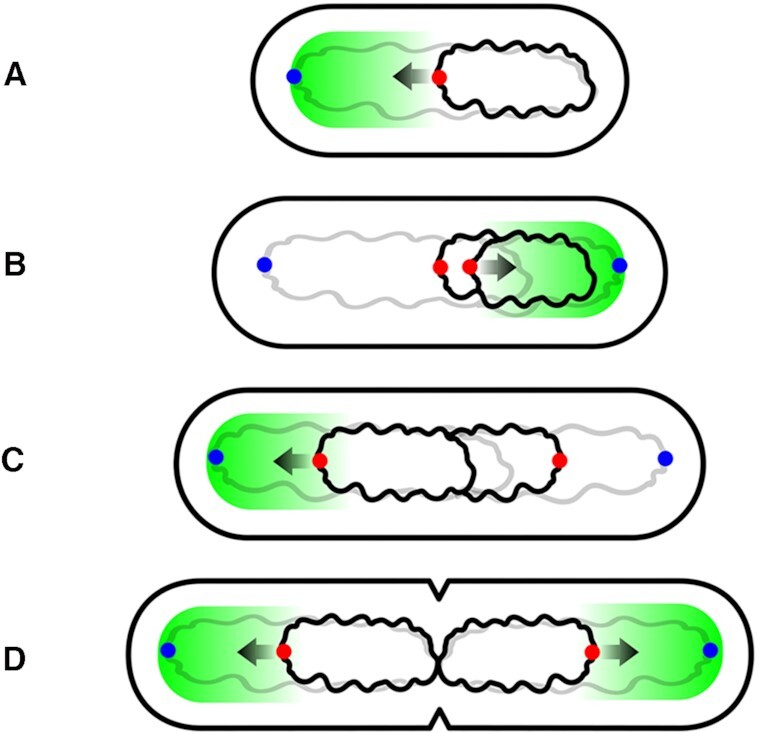
Tug-of-war model for *V. cholerae* Chr2 segregation by ParA2 oscillations. (**A**) In a young cell, ParA2 waves (green area) oscillate pole-to-pole that results in positioning ParB2-*parS2* centromere (red dots) of Chr2 (black) at midcell via transient tugging in either poleward direction (‘tug-of-war’ motion). Arrow shows direction of tugging of the Chr2 centromere (ParB2-*parS2*). (**B**) When Chr1 (grey) replication is two-thirds completed, Chr2 replication initiates and segregation of sister centromeres follows. (Chr1 centromeres (blue dots) are segregated to the poles much earlier). The doubling of Chr2 centromeres further depletes ParA2 at midcell and this helps to distribute ParA2 towards an elongating cell pole. (**C**) In older predivisional cells, the oscillating ParA2 wave tug the Chr2 sister centromeres alternatively to bidirectionally segregate them to ¼ and ¾ cell length positions. (**D**) Completion of segregation of sisters of both Chr1 and Chr2 allows septal ring formation at the cell centre and ParA2 waves re-establishes oscillations within each daughter cell compartment to position the Chr2 centromere at midcell.

Based on our biochemical characterization, we propose a tug-of-war model for *V. cholerae* ParA2 dynamic gradients and how this could be driving Chr2 movement and positioning (Figure 9). We found that DNA rebinding could be regulated by three factors: the remodeling to active ParA2*-ATP dimers that bind DNA, the nucleotide exchange rate and the suppression of DNA exchange by ParB2. Overall, the rate of DNA rebinding is directly correlated to the concentration of ParA2 or the concentration ratios of ParA2:ParB2 in the cell. In a young cell when *ori2* is at midcell, ParB2 binds specifically with high local concentrations onto *parS2* sites to form the partition complex (Figure 9A). Upon stimulation of ATP hydrolysis by ParB2-*parS2* locus, ParA2-ADP dimers are released from the partition complex and surrounding DNA. Prior to Chr2 replication, the persistent localization of ParB2-*parS2* at midcell depletes and suppresses ParA2 from rebinding DNA at the midcell region. The delayed ATP recovery from slow remodeling to ParA2* and slow nucleotide exchange inhibits DNA rebinding and promotes ParA2 distribution to the polar region. Once ParA2*-ATP dimers rebind the nucleoid, they cooperatively accumulate as oligomers, setting up a protein gradient that is highest towards the cell poles. As ParB2-*parS2* locus remains at mid-cell relative to the dynamic oscillating ParA2 wave, we speculate that in young cells, ParA2 wave cooperatively binds onto the nucleoid as dynamic oligomers and constantly sweeps from one pole to the opposite pole to ‘tug’ at ParB2-*parS2* locus via ParA2–ParB2 interactions. This ‘tug-of-war’ motion fine-tunes and maintains the locus at midcell position with each periodic oscillation. Once Chr2 replication initiation is licensed at two-thirds of Chr1 replication cycle (40), the doubling of ParB2-*parS2* loci activates further depletion of ParA2 at midcell, redistributing the ParA2 wave towards elongating cell poles (Figure 9B). In older predivisional cells, the oscillating ParA2 waves ‘tug’ at the sister loci to bidirectionally segregate to quarter-cell positions, followed by the progressive separation of the bulk of Chr2. The chromosome terminus is held in place at midcell by MatP-*matS* until unlinking of sister chromosomes and segregation leads to septal ring formation (Figure 9C) (70). ParA2 oscillation re-establishes between the two cell halves and repositions the chromosome loci back to midcell positions in each of the daughter cells (Figure 9D).

There are several factors that compound the spatiotemporal dynamics of ParA2 that is required for chromosome segregation compared to plasmid partition. First, Chr2 cargo is 10x larger than plasmids, making the large chromosomal cargo less diffusive, allowing for more directed mobility than smaller plasmids (36). Second, Chr2 replication and segregation cycle has to spatiotemporally coordinate with Chr1 replication and cell division cycles (39,40). Chr2 has then to progressively segregate towards quarter-cell positions before licensing of septation by SlmA (71). Third, as the partition complex drives the bulk segregation of Chr2, the underlying structure of the nucleoid scaffold is also getting remodeled as a result of concomitant replication and segregation of Chr1 and Chr2. We believe that the fast kinetics of ParA2 ATPase form the basis of the oscillating ParA2 waves that drive the symmetrical positioning and segregation of Chr2, as well as increase the dynamic response to coordinate with Chr1 segregation and cell division. It will be important to test how variations of the kinetic principles could determine spatiotemporal dynamics that regulate chromosome and plasmid segregation dynamics across different bacteria.

## DATA AVAILABILITY

The authors confirm that the data supporting the findings of this study are available within the article [and/or] its supplementary materials.

## Supplementary Material

gkad321_Supplemental_Files

## Notes

Present address: Ling Chin Hwang, Medical Technology Research Centre, School of Medicine, Anglia Ruskin University, Chelmsford CM1 1SQ, UK.
