# Supplementary material for: Kinetic principles of ParA2-ATP cycling guide dynamic subcellular localizations in Vibrio cholerae
Source: Nucleic Acids Res. 2023 May 4;51(11):5603–20. doi: 10.1093/nar/gkad321 (PMC10287910; doi:10.1093/nar/gkad321)
Supplement: gkad321_Supplemental_Files [file gkad321_supplemental_files.zip › Chodha_2022_KineticsVcParA2_NAR_Suppl-gkad321.pdf]

Supplementary Data for

Kinetic principles of ParA2-ATP cycling guide dynamic subcellular localizations in *Vibrio cholerae*

Satpal S. Chodha[1], Adam C. Brooks[1], Peter Davis[1], Revathy Ramachandran[2], Dhruba K Chattoraj[2], Ling Chin Hwang[1,3,*]

[1] Department of Molecular Biology and Biotechnology, University of Sheffield, Firth Court, Western Bank, Sheffield, S10 2TN, UK

[2] Basic Research Laboratory, Centre for Cancer Research, National Cancer Institute, National Institutes of Health, Bethesda, MD 20892-4260, USA

[3*] Medical Technology Research Centre, School of Medicine, Faculty of Health, Education, Medicine and Social care, Anglia Ruskin University, Chelmsford, UK

* To whom correspondence should be addressed. Tel: +44 (0)1245 683238; Email: ling.hwang@aru.ac.uk

Present Address: [Ling Chin Hwang], Medical Technology Research Centre, School of Medicine Faculty of Health, Education, Medicine and Social care, Anglia Ruskin University, Chelmsford, CM1 1SQ, UK

This PDF file includes:

- Supplementary text
- Figures S1 to S8
- Tables S1 to S2
- Legends for Movies S1 to S4
- SI References

Other supplementary materials for this manuscript include the following:

- Movies S1 to S4

Supplementary Information Text

Materials and Methods

Buffers

Buffer A: 50 mM Tris-HCl pH 7.5, 100 mM NaCl, 10 mM $MgCl_2$, 10% glycerol, 100 mg/ml BSA, and 1 mM DTT. **Buffer B**: 50 mM Tris-HCl pH 7.5, 150 mM NaCl, 5 mM $MgCl_2$. **Par Buffer**: 50 mM Tris (pH 7.5), 100 mM NaCl, 5 mM $MgCl_2$, 10% (v/v) glycerol, 1 mM DTT, 0.1 mg/ml α-casein.

ParA2 protein expression and purification

10 ml of LB medium with 50 µg/ml kanamycin (Kan) was inoculated with *E. coli* BL21(DE3) transformed with pSC01, and grown at 37°C overnight. Culture was added to 2x500 ml fresh LB/Kan and grown to an OD_{600} 0.55. Cultures were cooled to 25°C and expression was induced with 1 mM isopropyl β-D-thiogalactoside (IPTG) for 2 h at 25°C. Cells were harvested by centrifugation for 20 min at 4,000 g and stored at -80°C. Cells obtained from a 1 L culture were defrosted and suspended in 10 ml per gram of pellet of Tris buffer (50 mM Tris-HCl pH 8.0, 0.1 M NaCl), along with ½ protease inhibitor tablet (Roche) and 1 mg/ml of lysozyme. Cells were placed on ice and disrupted by sonication using a medium probe on a Soniprep 150 using 3 cycles of 20 s at 16-micron amplitude. Cell debris was removed by centrifugation at 72,000 g for 10 min at 24,500 rpm and the supernatant fraction was used for purification. ParA2 purification was performed on an FPLC AKTA system (GE Healthcare). The cell extract was applied on a 5 ml Heparin-HP cartridge (GE Healthcare) equilibrated in Tris buffer (50 mM Tris-HCl pH 8.0, 0.1 M NaCl). Protein sample was eluted by a 50 ml gradient of 0−0.5 M NaCl in Tris buffer and 2.5 ml fractions were collected. The main peak containing protein was eluted at 0.25 M NaCl and 3-4 peak fractions were combined for further purification by anion exchange chromatography and gel filtration. The protein sample was diluted 2.5-fold with water to 0.1 M NaCl and applied on a 6 ml Resource Q column (GE Healthcare) equilibrated with Tris buffer. Elution was performed at 6 ml/min with 60 ml gradient of 0.1−0.7 M NaCl and 2.5 ml fractions collected. ParA2 was eluted at 0.35 M NaCl. Two peak fractions were combined and concentrated to 1 ml (Vivaspin 50,000 MWCO) and loaded on a 1.6x60 HiLoad Superdex 200 column equilibrated in 0.5 M NaCl 50mM Tris-HCl pH 8.0. Gel filtration was performed at 1.5 ml/min flow rate. Peak fractions were combined and concentrated. DTT, EDTA and glycerol were added to final concentrations of 2 mM, 0.1 mM and 10%, respectively, before storage at -80°C until further use. SDS-PAGE showed ParA2 yields with 98% purity. Protein sequence of ParA2 was analyzed with mass spectrometry.

ParA2-GFP purification

10 ml of LB medium with 50 µg/ml ampicillin (Amp) was inoculated with *E. coli* BL21(DE3) transformed with pLCH02 and grown at 37°C overnight. Culture was added to 2 x 500 ml fresh LB/Amp and grown to an OD_{600} 0.5. Cultures were cooled to 16°C and expression was induced with 1 mM IPTG overnight at 16°C. Cells were harvested by centrifugation for 20 min at 4,000x*g* and stored at -80°C. Cells obtained from a 1 L culture were defrosted and suspended in 10 ml per gram of pellet of 50 mM Tris-HCl pH 8.0, 0.1 M NaCl, along with ½ a protease inhibitor tablet (Roche) and 1 mg/ml of lysozyme. Cells were placed on ice and disrupted by sonication on a Soniprep 150 machine. Three cycles of 20 s treatment at 16-micron amplitude were applied with cooling between the treatments. Cell debris was removed by centrifugation for 10 min at 24,500 rpm (72,000x*g*). The supernatant fraction (cell free extract, CFE) was separated and used for purification. CFE was applied on a 5 ml His-Trap HP column (GE Healthcare) equilibrated in 50 mM Tris-HCl pH 8.0, 0.1 M NaCl, with an AKTA purifier system with a flow rate of 5 ml/min. Bound protein was eluted by a 50 ml gradient of imidazole from 0 to 0.5 M in 50 mM Tris-HCl pH 8.0, 0.1 M NaCl. Peak fractions were combined, and the volume of the protein sample was reduced to <2 ml. Sample was applied to 1.6 x 60 ml HiLoad Superdex 200 column equilibrated in buffer A. Gel filtration was performed at 1.5 ml/min flow rate. 2 ml fractions were collected after the void volume. Peak fractions were combined and concentrated. 0.3 mg of TEV protease was added per 1 mg ParA2-GFP-His in 50 mM Tris pH 8.0, 150 mM NaCl and left overnight at 16°C.

Sample was suspended in 25 mM Tris pH 8.0, 500 mM NaCl, 20 mM imidazole, 10% glycerol and 2 mM BME and loaded onto a 5 ml HisTrap column, eluting over a 12 CV gradient. The flow through was collected and reloaded onto the column to run once more. Protein was eluted with 25 mM Tris pH 8.0, 500 mM NaCl, 1 M imidazole, 10% glycerol and 2 mM BME. Peak fractions were collected, concentrated to <2 ml and buffer exchanged to 50 mM Tris pH 8.0, 500 mM NaCl, 10% glycerol, 2 mM DTT, and 0.1 mM EDTA using a VivaSpin column. The sample was then loaded onto a Superdex 200 16/600 column and eluted over a 1.2 CV isocratic gradient. Peak fractions were pooled, concentrated, and stored at -80°C.

ParA2 K124R/Q/E purification

5 ml LB medium with 100 µg/ml ampicilin (Amp) was inoculated with *E. coli* BL21(DE3) transformed with pRCT01/02/03, and grown as the protocol for ParA2. This culture was added to 500 ml fresh LB/Amp to an OD_{600} 0.55. Cultures were cooled to 25°C and expression was induced with 1 mM IPTG for 2 h at 25°C. Cells were harvested by centrifugation for 20 min at 4,000x*g* and stored at -80°C. For each mutant, a 0.5 L culture pellet was thawed, and resuspended in 10 ml per gram of pellet in sonication buffer (50 mM Tris-HCl pH 8.0, 50 mM $(NH4)_2SO4$, 1 mM EDTA) along with ½ a protease inhibitor tablet (Roche) and 1 mg/ml of lysozyme. The cells were lysed by sonication for a total of 6 min at 30 s intervals at 12-micron amplitude and then centrifuged at 60,000x*g* at 4 °C for 25 min. Discarding the pellet, 0.35 g of ammonium sulphate was added per ml of supernatant before centrifugation at 60,000x*g* for 25 min at 4°C. The supernatant was discarded, and the pellet was resuspended (in 10 ml) and left to dialyse overnight against 50 mM Tris-HCl pH 8.0, 100 mM NaCl, 10% glycerol, 0.1 mM EDTA, 2 mM DTT. The cell free extract was loaded onto a 5 ml HiTrap Heparin column and eluted against 50 mM Tris-HCl pH 8.0, 1 M NaCl, 10% glycerol, 0.1 mM EDTA, 2 mM DTT over a 12 CV gradient (0.1 M NaCl to 1 M NaCl); pooled fractions were then loaded onto a 1 ml Mono Q column and eluted against 50 mM Tris-HCl pH 8.0, 1 M NaCl, 10% glycerol, 0.1 mM EDTA, 2 mM DTT over a 20 CV gradient. Peak fractions were pooled and concentrated to <2 ml, before loading onto a Superdex 200 16/600 and eluted against 1.2 CV of storage buffer (30 mM Tris-HCl pH 7.5, 0.5M NaCl, 10% glycerol, 0.1 mM EDTA, 2 mM DTT). Peak fractions were pooled, concentrated, and stored at -80 °C.

ParB2 purification

5 ml of LB medium with 100 µg/ml Amp was inoculated with *E. coli* BL21(DE3) transformed with pLCH04 and grown as in for ParA2. Culture was added to 500 ml fresh LB/Amp to an OD_{600} 0.55. Cultures were cooled to 25°C and expression was induced with 1 mM IPTG for 4 h at 25°C. Cells were harvested as described for ParA2. CFE was applied on a 5 ml His-Trap HP column (GE Healthcare) equilibrated in 50 mM Tris-HCl pH 8.0, 0.1 M NaCl at flow rate 5ml/min. Bound protein was eluted by a 50 ml gradient of imidazole from 0 to 0.35 M in 50 mM Tris-HCl pH 8.0, 0.1 M NaCl. Peak fractions were combined, and the volume of the protein sample was reduced to <2 ml (using Vivaspin 50,000 MWCO). Sample was applied to 1.6 x 60 ml HiLoad Superdex 200 column equilibrated in 0.5 M NaCl, 50mM Tris-HCl pH 8.0. Gel filtration was performed at 1.5 ml/min flow rate and 2 ml fractions were collected after void volume. Peak fractions were combined and concentrated using Vivaspin 50,000 MWCO. The TEV-cleavage protocol for ParB2-His was performed as for ParA2-GFP-His above. Protein was concentrated, and stored at -80 °C

FRAP experiments

A free-space 488 nm laser (Coherent) was aligned to a TIRF microscope via the backport for photobleaching. The laser beam was focused by a meniscus lens (f=-500 mm, Thorlabs) and reflected by a ZT488/640rpc dichroic mirror (Chroma) into the back of the objective. ParA2-GFP (10 µM) was preincubated in the presence of 2 mM ATP for 15 min at 25°C and diluted 10X before infusion to the flowcell at various densities. Camera acquisition was at 1 frame/s and exposure time was 100 ms. Acquisition was stopped for ~1 s as the DNA carpet was photobleached to around 50% of initial intensity, before continuing acquisition. FRAP curves were normalized and corrected for background intensity and overall background bleaching due to FRAP. Each curve was fitted to a double exponential equation in Origin Pro software.

Construction of strains for microscopy
A plasmid expressing ParA2-fused to GFP was electroporated into CVC209 (see Strain list) and used for microscopy as described below. First, the *parA2:gfp* DNA fragment was amplified from pLCH02 template DNA using primers LCH11-A2gfp-fwd and LCH12-A2gfp-rev. The DNA fragment was then inserted into pBAD-HisB to construct plasmid pLCH08. The ampicillin resistance gene cassette was interrupted by inserting a *kanr*2 DNA fragment (amplified from pACYC177) resulting in the kanamycin-resistant plasmid pLCH10.

Strain List:

Strain	Description	Reference	Used in Figure
CVC209	*V. cholerae* El Tor N16961; Str^R	(2)	Fig. 1

Plasmid List:

Plasmid	Description	Reference	Used in Figure
pLCH02	pET15b-*parA2-GFP*-his; Ap^R	This Study	Figures 1, 6
pLCH08	P_{BAD}-*parA2:GFP* in pBAD-HisB; pBR*ori*; Ap^R	This Study	Figure 1
pLCH10	P_{BAD}-*parA2:GFP* in pBAD-HisB; pBR*ori*; Kn^R	This Study	Figure 1
pLCH11	P_{BAD}-*parA2 K124R:GFP* in pBAD-HisB; pBR*ori*; Kn^R	This Study	Figure 6
pLCH12	P_{BAD}-*parA2 K124Q:GFP* in pBAD-HisB; pBR*ori*; Kn^R<br>Site specific mutagenesis of pLCH10	This Study	Figure 6
pLCH13	P_{BAD}-*parA2 K124E:GFP* in pBAD-HisB; pBR*ori*; Kn^R<br>Site specific mutagenesis of pLCH10	This Study	Figure 6
pRCT01	P_{BAD}-*parA2 K124Q* in pBAD-HisB; pBR*ori*; Ap^R	This Study	Figure 6
pRCT02	P_{BAD}-*parA2 K124E* in pBAD-HisB; pBR*ori*; Ap^R	This Study	Figure 6
pRCT03	P_{BAD}-*parA2 K124R* in pBAD-HisB; pBR*ori*; Ap^R	This Study	Figure 6
pSC01	*parA2* in pET28 b (+); Kn^R	This Study	Figures 2-5
pLCH04	*parB2-his* in pET15 b (+); Ap^R	This Study	Figures 2, 4, 6
pBKSII	pBluescriptII KS (+)	Stratagene	Figure 5

Oligonucleotide list:

Labeled nsDNA	DNA sequence
Cy3-69 bp nsDNA	Cy3-<br>GAGGTCGACGGTATCGATAAGCTTGATATCGAATTCCTGCAGCCCG<br>GGGGATCCACTAGTTCTAGAGC
Cy5-144 bp nsDNA	Cy5-<br>GTAAAACGACGGCCAGTGAGCGCGCGTAATACGACTCACTATAGGG<br>CGAATTGGAGCTCCACCGCGGTGGCGGCCGCTCTAGAACTAGTGGA<br>TCCCCCGGGCTGCAGGAATTCGATATCAAGCTTATCGATACCGTCGA<br>CCTCG

Primer List:

**Primer Name**	**Sequence**	**Used for amplifying**	**Used in Figure**
LCH11-A2gfp-fwd	ATACCATGGCAATGAAACG	*parA2:gfp* for pLCH02	Figure 1
LCH12-A2gfp-rev	ATAGAATTCTTAGCCCGAGTGATGG		Figure 1
SK-fwd	GCTCTAGAACTAGTGGATCC	Cy3-labeled 69 bp DNA from pBKSII	Figure 5
KS-rev-Cy3	Cy3-CGAGGTCGACGGTATCG		
M13-fwd-Cy5	Cy5-GTAAAACGACGGCCAGT	Cy5-labeled 144 bp DNA from pBKSII	Figure S5
KS-rev	CGAGGTCGACGGTATCG		
LCH19-ParA2-K124R-rev	CAGATGAACAGCCGTCATACTGCGGCC GGTACCGCCTTTTTGATT	Site specific mutagenesis of pLCH10 for pLCH11	Figure 6
LCH20-ParA2-K124R-fwd	AATCAAAAAGGCGGTACCGGCCGCAGT ATGACGGCTGTTCATCTG		Figure 6
LCH21-ParA2-K124Q-rev	ATGAACAGCCGTCATACTCTGGCCGGT ACCGCCTTTTTG	Site specific mutagenesis of pLCH10 for pLCH12	Figure 6
LCH22-ParA2-K124Q-fwd	CAAAAAGGCGGTACCGGCCAGAGTATG ACGGCTGTTCAT		Figure 6
LCH23-ParA2-K124E-rev	CAGCCGTCATACTTTCGCCGGTACCGC CT	Site specific mutagenesis of pLCH10 for pLCH13	Figure 6
LCH24-ParA2-K124E-fwd	AGGCGGTACCGGCGAAAGTATGACGGC TG		Figure 6
RCT01_A2_Rev_ EcoR1	CTAGAATTCTTAGCCCTGATTCAGAGAG	*parA2 K124Q* for pRCT01	Figure 6
		parA2 K124E for pRCT02	
		parA2 K124R for for pRCT03	

A

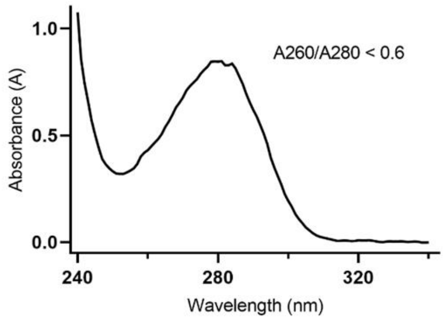

B

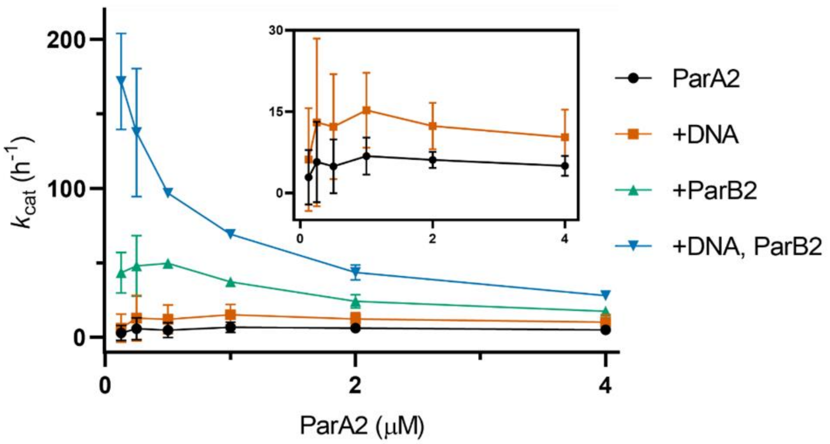

C

	ATPase	DNA	ParB	DNA, ParB	Reference
***Vc* ParA2**	6.15 ± 0.72	18.51 ± 0.76	27.83 ± 2.61	49.78 ± 3.36	This study
***Bs* Soj**	1.40	1.20	4.00	83.60	Scholefield et al., 2011
***Tt* Soj**	NA	0.27	1.27	2.00	Leonard et al., 2005
***Hp* Soj**	4.86	5.59	7.53	9.72	Lee et al., 2006
***Cc* ParA**	5.90	6.70	120*	-	Lim et al., 2014
	2.40	-	205.80	-	Easter & Gober, 2002
**P1 ParA**	1.13	1.57	4.71	12.53	Davis et al., 1992
**F SopA**	0.22	0.41	0.28	0.78	Watanabe et al., 1992
	0.14	0.28	0.45	12.50	Ah-seng et al., 2009

k_{cat} (h^{-1}) of chromosome and plasmid ParAs

*equimolar ParB had no effect on ATP turnover, K_{ParB} = 80 μM

Figure S1. Comparison of k_{cat} of *Vc* ParA2 with plasmid and chromosomal ParA homologs. **(A)** *Vc* ParA2 spectra. The baseline corrected A260/A280 ratio is less than 0.6 corresponding to nucleotide-free ParA2 after ion exchange and gel filtration purification steps. **(B)** Catalytic constant, k_{cat} of ParA2 alone and when stimulated by cofactors ParB and DNA (this study). **(C)** Table of k_{cat} (h^{-1}) values of *Vc* ParA2 and ParA homologs. Compared to other plasmid and chromosomal ParAs, *Vc* ParA2 shows relatively higher k_{cat} values throughout.

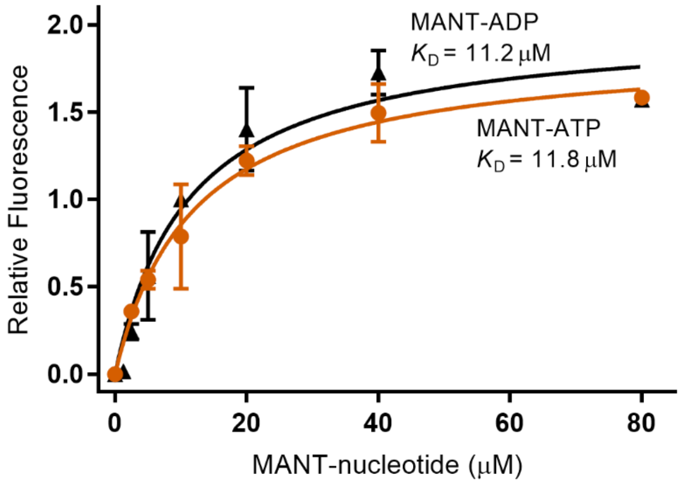

Figure S2. Binding curves of ParA2 and MANT-nucleotides. 1.5 μM ParA2 was mixed with indicated concentrations of MANT-ATP or MANT-ADP in Buffer B on ice. An initial fluorescence measurement was taken for each sample before incubating at 37°C for 20 min. The steady state fluorescence was then measured for each sample. Readings were acquired using a Fluorolog®-3 spectrofluorometer (Horiba Scientific) and a 'SpectraACQ' controller set at 356 nm ± 1.2 nm, in a 'HellmaAnalytics High Precision Cell'. Experiments were repeated two times. The relative fluorescence change was fitted in GraphPad Prism 8, with a saturation, one-site specific binding equation, to derive K_D.

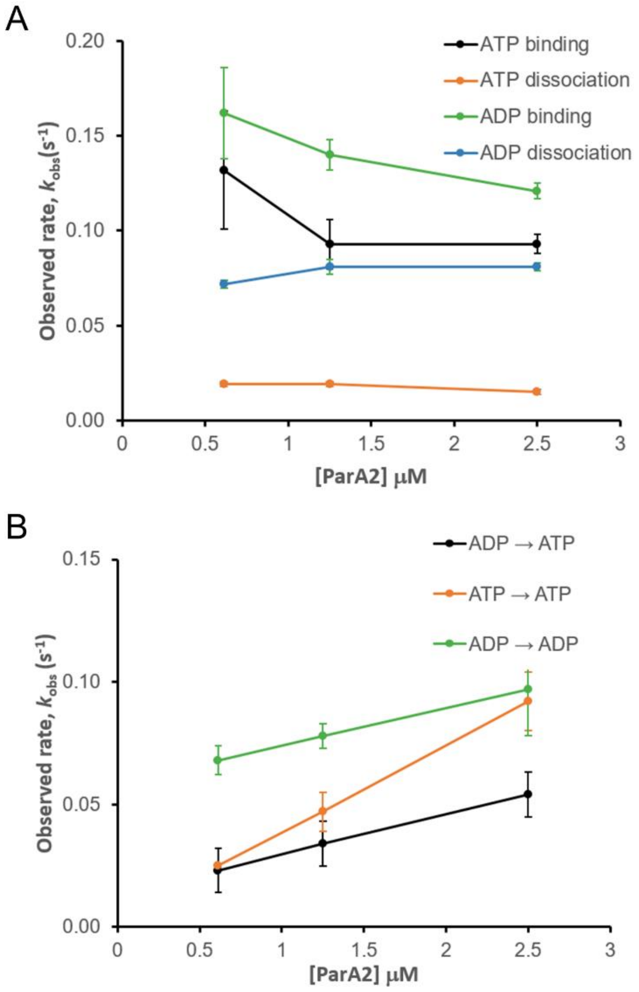

Figure S3. Stopped flow kinetics of ParA2 interactions with adenine nucleotides. (A) In nucleotide binding assays, 0.6, 1.25, 2.5 μM ParA2 were rapidly mixed with 25 μM MANT-AXP and fluorescence increase was monitored over time. The observed binding curves were fitted with single exponential increase to determine observed rates of binding, k_{obs} and plotted vs. ParA2 concentration. In nucleotide dissociation assays, 2.5 μM ParA2 and 5 μM MANT-AXP were pre-incubated at 23°C for 3 min, then rapidly mixed with 1 mM unlabeled AXP and their fluorescence decrease over time was fitted to obtain observed rates of dissociation, k_{obs}. **(B)** Nucleotide exchange assay with 0.625, 1.25, 2.5 μM ParA2 pre-incubated (at a 1:5 ratio) with 3.125, 6.25, 12.5 μM unlabeled AXP, respectively, then rapidly mixed with 15.625, 31.25, 62.5 μM MANT-AXP (at 5x higher concentrations than AXP). The fluorescence increase was fitted to obtain observed rates of nucleotide exchange, k_{obs}. All data are averages of at least two experiments.

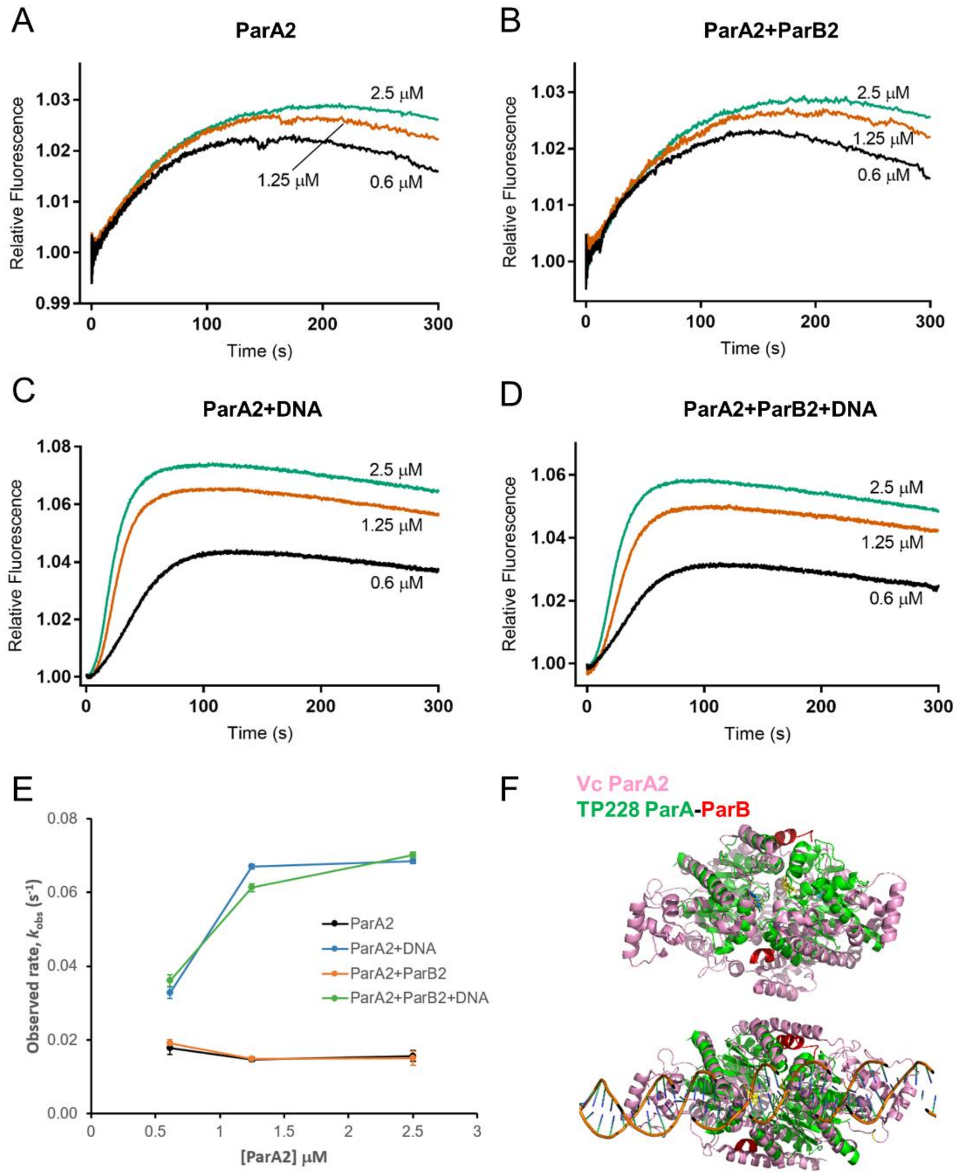

Figure S4. Stopped flow kinetics of ParA2 tryptophan fluorescence. (A) Premix 1 with indicated concentrations of ParA2 was rapidly mixed with premix 2 containing 1 mM ATP, both in Buffer B. Relative fluorescence increase was monitored over time. **(B)** ParA2 tryptophan fluorescence kinetics in the presence of ParB2. Same as in (A), except with 0.6 μM ParB2 added to premix 2. **(C)** ParA2 tryptophan fluorescence kinetics in the presence of DNA. Same as in (A), except with 0.1 mg/ml DNA added to premix 2. **(D)** ParA2 tryptophan fluorescence change in the presence of ParB2 and DNA. Same as in (A), except with 0.6 μM ParB2 and 0.1 mg/ml DNA added to premix 2. **(E)** Observed rates of relative fluorecence increase fitted from curves **(A)-(D)** show the highest increase with DNA, independent of ParB2. **(F)** Top: Overlay of Vc ParA2-ADP dimers (PDB: 7NPE) and TP228 ParA-AMPPNP-ParB dimers (5U1G). Bottom: Overlay of Vc ParA2-ATPγS dimers on DNA (7NPF) with TP228 ParA-AMPPNP-ParB dimers (5U1G) show ParB N-terminal helices (red) insertion in ParA dimer interface do not clash with DNA.

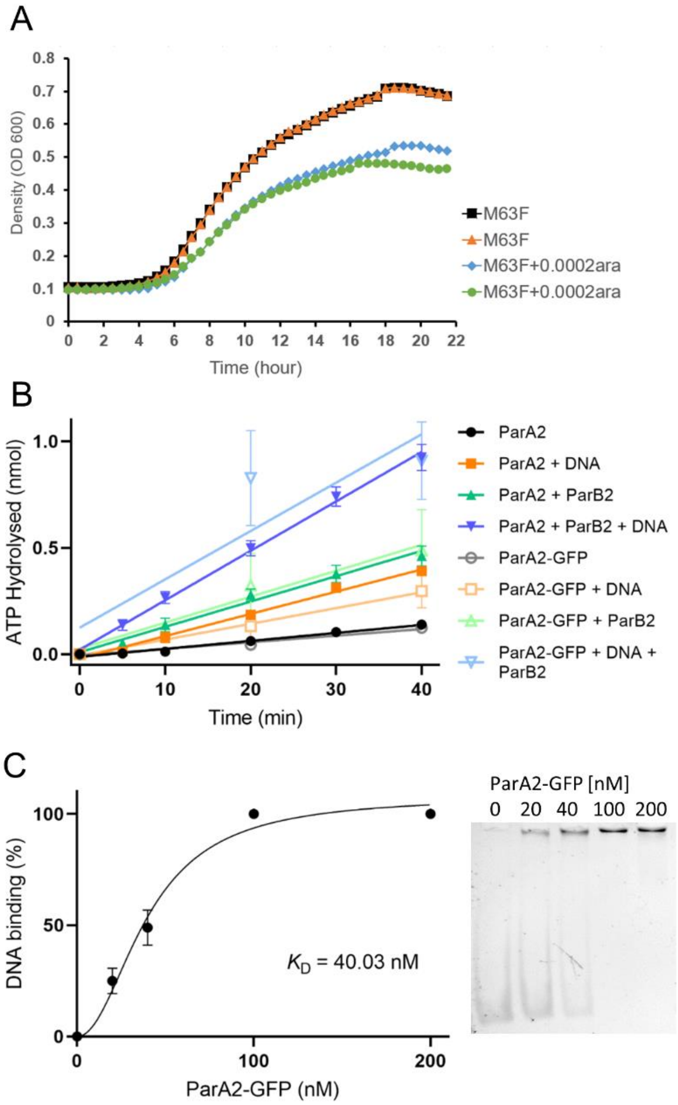

Figure S5: ParA2-GFP is mildly toxic to the growth of *V. cholerae* cells at low expression levels but is fully functional as the WT ParA2 *in vitro*. (A) Growth curves of *V. cholerae* cells. Cells were grown in M63 medium with WT ParA2 in 0.2% fructose (M63F) and ParA2-GFP expressed with 0.0002% arabinose (M63F+0.0002ara). Growth curves were recorded overnight (2 repeats). **(B)** ATPase assays of ParA2-GFP vs. WT ParA2. 1.5 µM ParA2 was mixed in Buffer A with 200 µM ATP spiked with 64 nM [α-^{32}P]-ATP. 1.5 µM ParB2 and 100 µg ml^{-1} sonicated salmon sperm DNA were added where indicated. The hydrolysis products were measured at 23°C after the indicated reaction times. The rate of ATP hydrolysis by ParA2-GFP was comparable to that of WT ParA2. Rates of ParB2 and/or DNA stimulated ATPase activities of ParA2-GFP and WT ParA2 are similar. **(C)** EMSA of ParA2-GFP showed cooperative binding to DNA. ParA2-GFP was titrated at increasing concentrations with 5 nM Cy3-labeled 69 bp DNA in Buffer A, in the presence of 2 mM ATP. One of three repeat experiments is shown here. The samples were analyzed by 5% PAGE in TBM buffer. ParA2-GFP-DNA binding affinity K_D 40 nM (left) was similar to wild-type ParA2 K_D 46 nM (Fig. 6C). These data indicate that purified ParA2-GFP retains the biochemical properties of WT ParA2.

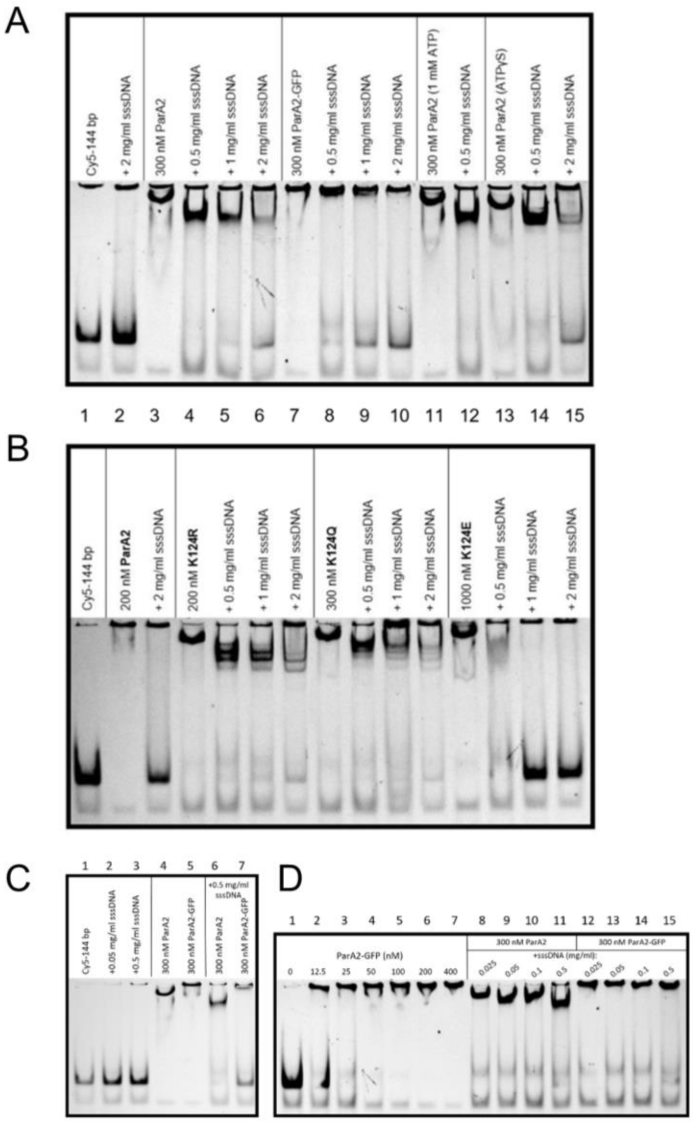

Figure S6. Dissociation of ParA2 and its K124 variants from DNA. (A) EMSA of ParA2-DNA complexes formed by incubation with 300 nM ParA2, 5 nM Cy5-144 bp DNA, 2 mM ATP (lanes 3, 7, 11, 13) unless stated otherwise. Controls of Cy5-DNA and with sssDNA (lanes 1, 2) showed a dark band with fainter lower band at the bottom of gel, deemed as a by-product of PCR. Addition of increasing concentrations of sssDNA (lanes 4-6) shows competition with bound DNA substrate (upper band) and increased dissociation of complexes to free DNA (lower band). Complexes formed with ParA2-GFP-His (lanes 7-10) showed similar levels of dissociation as wt ParA2 upon addition of sssDNA. Lowering ATP concentrations to 1 mM (lanes 11, 12) showed similar level of dissociation as was seen with the higher ATP concentration (2 mM) (lanes 3, 4). Reactions incubated with 1 mM ATPγS (lanes 13-15) showed similar levels of DNA dissociation as for complexes with ATP (lanes 3, 4, 6). **(B)** ParA2 K124 variants binding to and dissociation from DNA. Experimental set up is as in (**A**). K124R and K124Q bound DNA but dissociated from DNA to a lesser extent than WT ParA2. Complex formation with K124E required a much higher concentration of the protein but the complex dissociated with half the amount of competing DNA (lane 14). **(C)** Comparisons of ParA2 and ParA2-GFP binding to and dissociation from Cy5 144bp-DNA. Reactions were prepared as in (A). **(D)** Repeat of experiments as in (C) with different concentrations of competing sssDNA.

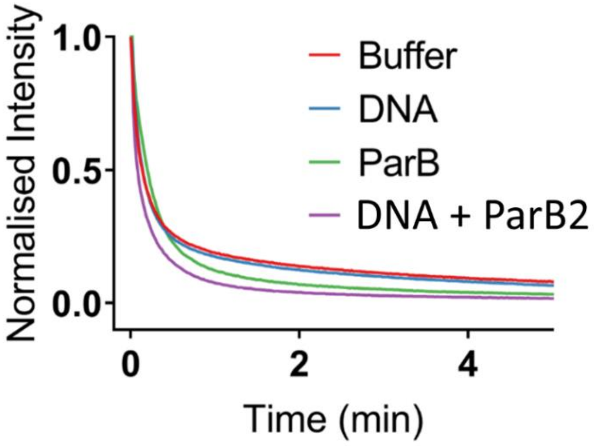

	Buffer	DNA	ParB2	DNA+ParB2
**K_1 (s^{-1})**	0.11	0.10	0.13	0.15
**K_2 (s^{-1})**	0.003	0.003	0.005	0.010
**τ_1 (s)**	9.3	9.9	7.6	6.7
**τ_2 (s)**	324	305	213	98
**$\%_1$**	78.6	80.5	74.3	83.1

K_1, τ_1 and $\%_1$: Dissociation rate, time constant and fraction of fast decay species, respectively.
K_2, τ_2: Dissociation rate and time constant of slow decay species, respectively.

Figure S7. Dissociation of ParA2-GFP from DNA carpet in the presence of cofactors. Buffer containing 1 µM ParA2-GFP preincubated with ATP was infused into a DNA carpeted flowcell until the binding reached steady state. Buffer flow was switched at t=0 to a wash buffer either alone, or with 100 µg/ml DNA or 2 µM ParB2, or with 100 µg/ml DNA and 2 µM ParB2. Fluorescence intensity was measured over time. Fluorescence intensities were subtracted for background and normalized to their prewash levels. The dissociation curves were fitted to a two-exponential decay model using GraphPad Prism 8.3 software and the fitted parameters are listed in table below. The cofactors ParB2 and DNA appeared to have a minor increase in rates of dissociation of ParA2-GFP complexes and the significance of the differences, if any, remains to be established.

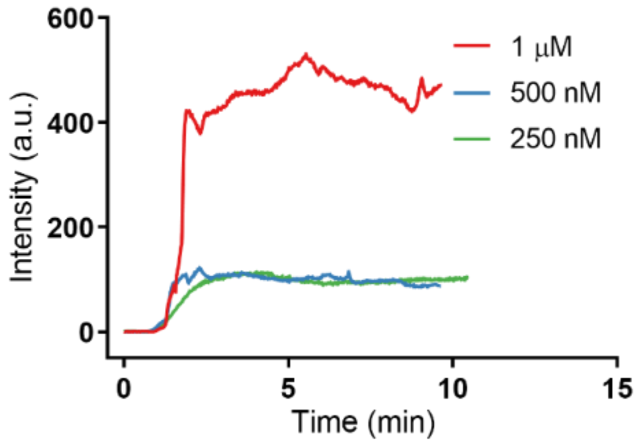

[ParA2]	1 μM	500 nM	250 nM
K (s^{-1})	0.0148	0.0347	0.0192
τ (s)	67.6	28.8	52.0

Figure S8. ATP start assay of ParA2-GFP. Solutions of 2, 1, and 0.5 µM ParA2-GFP and 2 mM ATP were prepared separately in Par Buffer. The ParA2-GFP and ATP solutions were loaded into 1 ml syringes and attached to a micro-static T-mixer (Upchurch). The T-mixer was attached to the single inlet port of a DNA-carpeted flowcell. Both samples were infused at 10 µl/min each with a combined flowrate of 20 µl/min entering the flowcell. Final concentrations of ParA2-GFP after mixing are as indicated. The flowcell was imaged with a TIRF microscope close to the inlet port to minimize the time between the point of solution mixing and protein binding. Fluorescence intensity of ParA2-GFP plotted vs. time of sample infusion showed a lag time of ParA2-GFP binding to the DNA carpet and lower intensities compared to ParA2-GFP preincubated with ATP (Figure 6B). Binding curves were fitted to single exponential increase. The Table shows fitted time constants and observed rates of DNA binding for each protein concentration.

Table S1. Binding and dissociation rates of ParA2-GFP on DNA carpet

ParA2-GFP	1 μM (ATP)	500 nM (ATP)	250 nM (ATP)	1 μM (ATPγS)
**Binding**				
**K (s^{-1})**	0.053 ± 0.006	0.051 ± 0.009	0.049 ± 0.011	0.016 ± 0.004
**τ (s)**	18.8 ± 1.9	20.0 ± 3.4	21.1 ± 4.4	64.5 ± 14.6
**Dissociation**				
**K_1 (s^{-1})**	0.11 ± 0.04	0.17 ± 0.08	0.19 ± 0.12	0.015 ± 0.002
**$τ_1$ (s)**	10.0 ± 4.2	7.2 ± 4.5	7.0 ± 4.3	70.2 ± 9.7
**K_2 (s^{-1})**	0.0046 ± 0.0001	0.0048 ± 0.0001	0.0041 ± 0.0001	0.0016 ± 0.0002
**$τ_2$ (s)**	218 ± 6	208 ± 6	242 ± 6	627 ± 6
**$\%_1$**	78.6 ± 4.1	79.7 ± 5.0	83.2 ± 1.3	48.7 ± 3.4

K_1, **$τ_1$**, **$\%_1$**: Rate, time constant and fraction of decay of fast decay species, respectively.
K_2, **$τ_2$**: Rate and time constants of slow decay species.

Mean ± SD values are from three repeats of FRAP measurements.

Table S2. Fitted FRAP recovery time-onstants and fractions of ParA2-GFP on DNA carpet

	Density (%)	$τ_1$ (s)	$τ_2$ (s)	F_1	F_2	$F_{immobile}$
**ParA2**	28	2.3 ± 0.4	121 ± 32	0.64	0.23	0.13
	100	8.5 ± 1.8	181 ± 16	0.44	0.32	0.24
**ParA2:ParB2 (1:0.5)**	34	6.2 ± 0.5	241 ± 28	0.62	0.21	0.17
**ParA2:ParB2 (1:1)**	45	6.6 ± 1.5	274 ± 78	0.47	0.18	0.38
**ParA2:ParB2 (1:2)**	43	8.2 ± 1.7	308 ± 81	0.48	0.18	0.34

Density: Percentage of protein coverage relative to intensity of 1 μM ParA2-GFP on DNA carpet at steady state.
$τ_1$, **F_1**: Recovery time-constant and fraction of fast species, respectively
$τ_2$, **F_2**: Recovery time-constant and fraction of slow species, respectively
$F_{immobile}$: Fraction of immobile species that did not recover after post-bleach, $1-(F_1+F_2)$

Mean ± SD values are from three repeats of FRAP measurements.

Movie S1. ParA2-GFP pole-to-pole oscillations in live *Vibrio cholerae* cells.
ParA2-GFP expression was induced by 0.0008% arabinose for 1 hour at 30°C with shaking before imaging. Cells were imaged in the same medium and supplemented with an agarose pad containing 0.02% arabinose. Data acquisition rate was 2 frames per min at 200 ms exposure time. Movie playback is for 20 fps.

Movie S2. Binding and dissociation of ParA2-GFP on DNA carpet.
Final concentration of 1 µM ParA2-GFP preincubated with 1 mM ATP was infused into the DNA-carpeted flowcell and imaged with a TIRF microscope. Increase of fluorescence intensity shows ParA2-GFP binding to the DNA carpet. Flow of ParA2-GFP was switched to wash buffer at 26 s (in movie). Decrease of fluorescence intensity shows ParA2-GFP dissociation from the carpet. Data acquisition rate was 1 fps at 100 ms exposure time. Movie playback is for 20 fps.

Movie S3. FRAP of ParA2-GFP bound to DNA carpet at high density
Final concentration of 1 µM ParA2-GFP preincubated with 1 mM ATP was infused into the DNA-carpeted flowcell until the ParA2-GFP binding reached steady state (100% density). The ParA2-GFP-coated DNA carpet was photobleached at 1 s (in movie) and fluorescence recovery monitored with TIRF microscope. Data acquisition rate was 1 fps at 100 ms exposure time. Movie playback is for 20 fps.

Movie S4. FRAP of ParA2-GFP bound to DNA carpet at low density
Final concentration of 1 µM ParA2-GFP preincubated with 1 mM ATP was infused into the DNA-carpeted flowcell for a shorter time before reaching steady state (28% density). The ParA2-GFP-coated DNA carpet was photobleached at 1 s (in movie) and fluorescence recovery monitored with TIRF microscope. Data acquisition rate was 1 fps at 100 ms exposure time. Movie playback is for 20 fps.

SI References

1. Scholefield,G., Whiting,R., Errington,J. and Murray,H. (2011) Spo0J regulates the oligomeric state of Soj to trigger its switch from an activator to an inhibitor of DNA replication initiation. Mol. Microbiol., 79, 1089–1100.
2. Leonard,T.A., Butler,P.J. and Lowe,J. (2005) Bacterial chromosome ¨ segregation: structure and DNA binding of the Soj dimer — a conserved biological switch. EMBO J., 24, 270–282.
3. Lee,P.S. and Grossman,A.D. (2006) The chromosome partitioning proteins Soj (ParA) and Spo0J (ParB) contribute to accurate chromosome partitioning, separation of replicated sister origins, and regulation of replication initiation in Bacillus subtilis. Mol. Microbiol., 60, 853–869.
4. Lim,H.C., Surovtsev,I.V., Beltran,B.G., Huang,F., Bewersdorf,J. and Jacobs-Wagner,C. (2014) Evidence for a DNA-relay mechanism in ParABS-mediated chromosome segregation. Elife, 3:e02758.
5. Easter,J. and Gober,J.W. (2002) ParB-stimulated nucleotide exchange regulates a switch in functionally distinct ParA activities. Mol. Cell, 10, 427–434.
6. Watanabe,E., Wachi,M., Yamasaki,M. and Nagai,K. (1992) ATPase activity of SopA, a protein essential for active partitioning of F plasmid. Mol. Gen. Genet., 234, 346–352.
7. Davis,M.A., Martin,K.A. and Austin,S.J. (1992) Biochemical activities of the ParA partition protein of the P1 plasmid. Mol. Microbiol., 6, 1141–1147. 69.
8. Ah-Seng,Y., Lopez,F., Pasta,F., Lane,D. and Bouet,J.-Y. (2009) Dual role of DNA in regulating ATP hydrolysis by the SopA partition protein. J. Biol. Chem., 284, 30067–30075.
9. Hulme, E.C. and Trevethick M. A. (2010) Ligand binding assays at equilibrium: validation and interpretation. *British Journal of Pharmacology*, 161, 1219–1237.
10. J. F. Heidelberg, *et al.* (2000) DNA sequence of both chromosomes of the cholera pathogen Vibrio cholerae. *Nature* 406, 477–483.
